# A need for implementation science to optimise the use of evidence-based interventions in HIV care: A systematic literature review

**DOI:** 10.1371/journal.pone.0220060

**Published:** 2019-08-19

**Authors:** Joseph Cox, Cassidy Gutner, Nadine Kronfli, Anna Lawson, Michele Robbins, Lisette Nientker, Amrita Ostawal, Tristan Barber, Davide Croce, David Hardy, Heiko Jessen, Christine Katlama, Josep Mallolas, Giuliano Rizzardini, Keith Alcorn, Michael Wohlfeiler, Eric Le Fevre

**Affiliations:** 1 Division of Infectious Diseases, Department of Medicine, McGill University Health Centre, Montreal, Canada; 2 ViiV Healthcare, London, England, United Kingdom; 3 Pharmerit International, Rotterdam, the Netherlands; 4 Pharmerit International, Berlin, Germany; 5 Chelsea and Westminster Hospital, London, England, United Kingdom; 6 Università Cattaneo LIUC, Castellanza, Italy; 7 Whitman-Walker Centre, Washington, DC, United States of America; 8 Praxis Jessen, Berlin, Germany; 9 Hôspital Universitaire Pitié-Salpêtrière, Paris, France; 10 Hospital Clinic, Barcelona, Spain; 11 Ospedale Sacco, Milan, Italy; 12 NAM publications, London, England, United Kingdom; 13 AHF Clinics, Los Angeles/Miami, United States of America; University of Mississippi Medical Center, UNITED STATES

## Abstract

To improve health outcomes in people living with HIV, adoption of evidence-based interventions (EBIs) using effective and transferable implementation strategies to optimise the delivery of healthcare is needed. ViiV Healthcare’s Positive Pathways initiative was established to support the UNAIDS 90-90-90 goals. A compendium of EBIs was developed to address gaps within the HIV care continuum, yet it was unknown whether efforts existed to adapt and implement these EBIs across diverse clinical contexts. Therefore, this review sought to report on the use of implementation science in adapting HIV continuum of care EBIs. A systematic literature review was undertaken to summarise the evaluation of implementation and effectiveness outcomes, and report on the use of implementation science in HIV care. Ten databases were reviewed to identify studies (time-period: 2013–2018; geographic scope: United States, United Kingdom, France, Germany, Italy, Spain, Canada, Australia and Europe; English only publications). Studies were included if they reported on people living with HIV or those at risk of acquiring HIV and used interventions consistent with the EBIs. A broad range of study designs and methods were searched, including hybrid designs. Overall, 118 publications covering 225 interventions consistent with the EBIs were identified. These interventions were evaluated on implementation (N = 183), effectiveness (N = 81), or both outcomes (N = 39). High variability in the methodological approaches was observed. Implementation outcomes were frequently evaluated but use of theoretical frameworks was limited (N = 13). Evaluations undertaken to assess effectiveness were inconsistent, resulting in a range of measures. This review revealed extensive reporting on implementation science as defined using evaluation outcomes. However, high variability was observed in how implementation outcomes and effectiveness were defined, quantified, and reported. A more specific and consistent approach to conducting and reporting on implementation science in HIV could facilitate achievement of UNAIDS 90-90-90 targets.

## Introduction

To accelerate progress toward ending acquired immunodeficiency syndrome (AIDS) as a public health threat by 2030, the Joint United Nations Program on HIV/AIDS (UNAIDS) established the 90-90-90 targets [1]. These ambitious targets aim to diagnose 90% of all people living with HIV (PLHIV), provide antiretroviral therapy (ART) for 90% of those diagnosed and achieve virological suppression in 90% of those treated with ART by 2020. In 2017, an estimated 75% of PLHIV knew their HIV-positive status, of which an estimated 79% were receiving ART among whom 81% were virologically suppressed [1]. Recent epidemiological estimates and programme data from 168 countries in all regions reveal progress but persistent gaps across the HIV care continuum remain [1, 2]. The HIV care continuum constitutes sequential steps of medical care from HIV awareness and prevention to the achievement of virological suppression [2]. To achieve virological suppression, PLHIV need to know their HIV-infection status, be linked and engaged in care, and receive and adhere to the prescribed ART regimen. Effective evidence-based interventions (EBIs) are available for all steps along the HIV care continuum and have been implemented in different geographic settings and contexts with success [2]. Despite a global downward trend in the epidemic, progress along the continuum is variable and several regions are experiencing increases in new infections and a lack of progress toward the UNAIDS 90-90-90 targets [1]. Globally, as of 2017 only 47% of all PLHIV achieved virological suppression, which is far lower than the target of 73%, suggesting many regions are not on track to meet the 2020 target [1].

In order to support the UNAIDS 90-90-90 initiative across diverse contexts, it is essential to identify appropriate EBIs (i.e. relevant for settings given local epidemiology and health infrastructure), understand which EBIs are effective and how these can be implemented, scaled and replicated from single trials of local innovations to broad-scale use [3–5]. This is a recognized goal of implementation science [6]. Poorly specified and evaluated implementation strategies present challenges to those who seek to reproduce or scale up the intervention in different settings and contexts and potentially impede real-world adoption of the EBIs [6].

**Fig 1:**
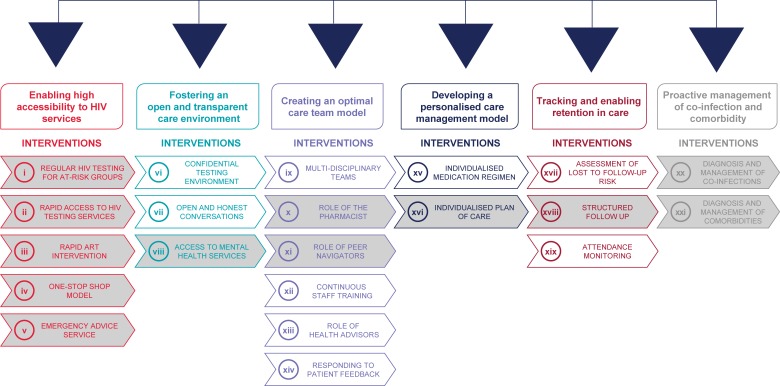
Positive pathways initiative: Compendium of 21 EBIs With 12 prioritised EBIs. From the compendium of 21 interventions, 12 were prioritized by an expert panel across six key themes of current HIV practice (interventions shaded under each of the six themes). Prioritization was based on a consideration of feasibility/perceived ease for care centres to trial the EBI. These EBIs are expected to be more widely used, investigated and reported. These 12 EBIs from the Positive Pathways initiative were included in the scope of the review. For details on the development of the compendium, refer to S1 Fig. ART, antiretroviral therapy; EBI, evidence-based intervention; HIV, human immunodeficiency virus; PrEP, pre-exposure prophylaxis.

ViiV Healthcare’s Positive Pathways initiative was developed with the overall objective to support the achievement of the UNAIDS 90-90-90 targets [7], by understanding current evidence-based practice in HIV care in real-world settings [7, 8]. This initiative set out to identify current EBIs along the HIV care continuum in centres across multiple geographies. The first phase of the Positive Pathways initiative was to map EBIs in high-income countries and develop a compendium of EBIs across the HIV care continuum as well as a self-assessment questionnaire. EBIs were thematically grouped and prioritised in terms of potential impact and practicality. A final compendium of 21 EBIs across six key themes of current HIV practice was established (Fig 1; for more details on the development of the Positive Pathways initiative refer to S1 Fig). The aim was to share the compendium and questionnaire with other HIV care centres to support the delivery of EBIs to increase prevention, diagnosis, linkage to care and retention in care.

During the development stage of the Positive Pathways initiative, it became apparent that effective knowledge transfer to share and embed EBIs in real-world settings would benefit from an implementation science approach. Implementation science is defined in the HIV Lancet as a ‘multidisciplinary specialty that seeks generalisable knowledge about the behaviour of stakeholders, organisations, communities, and individuals to understand the scale of, reasons for, and strategies to close the gap between evidence and routine practice for health in real-world contexts’ [9].

Before expanding the Positive Pathways initiative to other geographical regions, we sought to understand the extent to which identified EBIs were evaluated using implementation science within the targeted geographical area. To better understand the current use of implementation science in HIV care, only a selected set of EBIs were considered for this review. From the compendium of 21 EBIs, 12 were prioritized by an expert panel across six key themes of current HIV practice (interventions shaded under each of the six themes). Prioritization was based on a consideration of feasibility/perceived ease for care centres to trial the EBI. These 12 EBIs are expected to be more widely used, investigated and reported (Fig 1).

This information was considered instrumental in engaging care centres in the choice and adaptation of EBIs to their respective settings. Practically, given the focus on evaluation, studies using implementation outcome measures and related study designs may provide a broad appreciation for the use of implementation science in HIV care. Also, as implementation outcomes are key intermediate results in relation to clinical effectiveness, measures of effectiveness are also important to considering the use of implementation science.

Therefore, using measures and methods aligned with implementation science, we set out to summarise and critically appraise the evidence to obtain a better understanding of the current state of implementation science in HIV in high-income countries.

## Methods

The review focused on 12 EBIs (Fig 1; refer to S1 Fig for more information) and was conducted according to guidelines in the Cochrane Handbook for Systematic Reviews of Interventions [10] and Preferred Reporting Items for Systematic Literature Reviews and Meta-Analyses (PRISMA) [11] to obtain relevant information using a reproducible, robust and transparent methodology. In-line with these guidelines, we developed a study protocol in which the search strategy and study eligibility criteria were established prior to conducting the review. After this, searches were performed and retrieved publications were assessed for eligibility in a two-phase screening process based on predefined eligibility criteria. From the final list of publications considered relevant for this review, addata were extracted, the scope of which was also established *a priori*. As the final step, we synthesised key findings from the data. The review methodology is detailed below.

### Search sources and strategy

Given the objective of this review, we searched the following 10 databases: Medline, Embase, ABI/INFORM, Adis Pharmacoeconomic & Outcomes News, Allied and Complementary Medicine, DH-DATA: Health Administration Medical Toxicology and Environmental Health, Gale Group Health Periodicals Database, *Lancet* titles, *New England Journal of Medicine*, and Cochrane Central Register of Controlled Trials [12–21]. In the literature search strategy, we included both free-text and Emtree/MeSH terms for HIV, the 12 EBIs (Fig 1) and implementation outcomes. The search terms for implementation science were identified from previously published literature [6, 22, 23]. Hickey et al. [6] was used as the basis for the development phase of this study whereas Proctor et al. [23] was used to derive relevant implementation and service outcome search terms. This taxonomy [23] was chosen to guide the review as it is a widely used evaluation framework in the field of implementation science. Also, given the clear link between these outcomes and the evaluation of implementation strategies, the review was expected to be sensitive to detecting any research potentially aligned with implementation science. Also, Curran et al. [22] was used to identify relevant study designs for inclusion. As this review focused both on implementation and service (e.g. effectiveness) outcomes, we considered it appropriate to include hybrid study designs to be able to capture relevant publications for this review [22].

We searched the databases simultaneously via ProQuest [24], with the exclusion of the Cochrane Central Register of Controlled Trials Database which was searched via the Cochrane Library [25] (Search date: 29 March 2018). We applied different limits to the searches. This included restricting the geographical scope of the review to studies conducted in the United States, United Kingdom, France, Germany, Italy Spain, Canada, Australia, and Europe. This was done because the EBIs resulting from the Positive Pathways initiative involved only high-income countries. Furthermore, the review was restricted to English only publications and publication year from 2013 to 2018. Considering the fact that implementation science is an emerging field within HIV with guidance published in 2011 and 2012 to advance the understanding of implementation science [22, 23], the search timeframe of five years was deemed appropriate by the authors to identify relevant publications (for full details on the search strategy refer to S1 Table).

### Inclusion and exclusion criteria

Eligibility criteria for this review are provided in Table 1. In this review, publications were eligible if they reported on PLHIV or individuals at risk of being infected with HIV and who received an intervention that could be categorised into the 12 EBIs (Fig 1; refer to S1 Fig for more information). For inclusion it was required that the publication reported on outcomes related to the implementation, effectiveness or both, of the intervention. Including both implementation and effectiveness outcomes as per Proctor and colleagues [23] allowed for a review that was comprehensive in scope and could produce the most accurate overview of implementation science in the targeted countries. Implementation outcomes were determined using Proctor et al.’s taxonomy [23]. The effectiveness outcomes of interest (i.e. linkage to care, retention in care and medication adherence) were chosen because of their key role in achieving the UNAIDS 90-90-90 targets. We considered a broad range of study designs for inclusion including observational and experimental study designs (such as randomised controlled trial [RCT]), qualitative study designs (such as focus groups and interviews) as well as hybrid study designs which combine attributes of both quantitative and qualitative data collection.

**Table 1:** Inclusion and exclusion criteria.

	Inclusion Criteria	Exclusion Criteria
**Population**	Human beings infected by HIV Human beings having AIDS Human beings with high risk of being infected by HIV	Subjects are not human beings Subjects do not have HIV/AIDS Subjects not having high risk of being infected by HIV
**Intervention**	The 12 prioritised EBIs of Positive Pathways initiative: • Regular HIV testing for at risk groups • Rapid access to testing services • Rapid ART intervention • One stop shop model • Emergency advice service • Access to mental health services • Role of the pharmacist • Role of the care navigators • Individualised plan of care • Structured follow-up • Diagnosis & management of co-infections & co-morbidities	Interventions not according to the inclusion criteria, for example: • HIV/AIDS treatment being investigated only in the clinical trial setting • HIV/AIDS management model only been developed theoretically and not yet implemented in the real-world setting
**Outcome**	Implementation science outcomes from Proctor et al [23]: • Adoption • Acceptability • Appropriateness • Feasibility • Fidelity • Penetration • Sustainability • Implementation costs Other outcomes with regard to effectiveness of the interventions, such as: • Linkage to care • Adherence • Retention to care	Outcomes other than those defined in the inclusion criteria
**Study Design**	Review paper Quantitative studies and qualitative studies (such as RCTs and observational studies) Hybrid (type 1 and type 2) studies from Curran et al. [22]	Meta-analysis Letter to editor Newspaper Editorial Comment Opinion paper
**Geographic Scope**	Europe (continent), EU5 (UK, Germany, France, Italy, Spain), USA, Canada, Australia	Areas other than those specified in the inclusion criteria

AIDS, acquired immunodeficiency syndrome; ART, antiretroviral therapy; EBI, evidence-based intervention; HIV, human immunodeficiency virus; RCT, randomised controlled trial.

### Screening and selection

After the searches were performed, identified publications were screened in two phases, with reviews divided between three reviewers (LN, MB and NB). The first phase included screening of titles and abstracts of all publications based on the eligibility criteria followed by a second phase which included reviewing the full-texts of articles using the same criteria (Table 1).

### Data extraction and descriptive analyses

After we identified the eligible publications for this review, one reviewer (AO, NB, MB) extracted the relevant data from these publications. A second reviewer (LN) quality checked the data extracted. Discrepancies were resolved through discussion and consensus between the reviewers. We determined data extraction parameters *a priori* and included intervention details (e.g. type of intervention, including category of EBI, location of intervention, target population), implementation outcomes (e.g. parameter assessed, methodology of assessment, use of a theoretical framework and reported values of the parameter assessed) and effectiveness outcomes (i.e. linkage to care, retention to care and medication adherence). Transferable implementation strategies are required to ensure the consistent use of evidence to change healthcare policy and practice, therefore theoretical frameworks play a key role in implementation science [26, 27]. Given the number of theoretical frameworks available to evaluate the implementation of EBIs, we considered it most appropriate to focus on the taxonomy of implementation outcomes as defined by Proctor et al. [23]. However, we included theoretical frameworks as a relevant parameter for data extraction.

In this paper we provide a descriptive overview of the types of EBIs identified using the extracted dataset. This is followed by a discussion on the distribution of EBIs across three possible categories of evaluation: evaluation of both implementation and effectiveness, evaluation of implementation, and evaluation of effectiveness. Also, we provide a description on the implementation outcomes and effectiveness outcomes that have been documented across the 12 EBI categories. Evaluation of the EBIs using implementation and effectiveness outcomes are reported separately in the results section. As this review aims to document and better understand the current state of implementation science in HIV, our results focus primarily on the identification of documented implementation and service outcomes and the methodologies used to evaluate the implementation of EBIs. As implementation outcomes are key intermediate results in relation to clinical effectiveness, effectiveness outcomes were included in this review as secondary outcomes of interest. The description on effectiveness outcomes only focuses on the identification of documented effectiveness outcomes.

## Results

### Included studies

**Fig 2:**
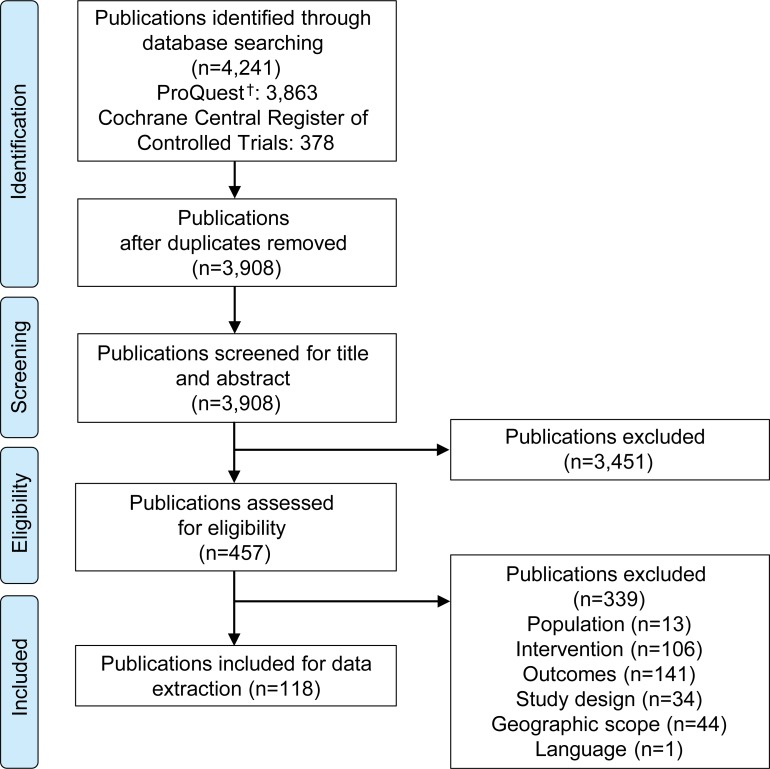
PRISMA flow diagram. PRISMA, Preferred Reporting Items for Systematic Literature Reviews and Meta-Analyses. ^†^Comprises the records identified via Medline, Embase, ABI/INFORM, Adis Pharmacoeconomic & Outcomes News, Allied and Complementary Medicine, DH-DATA: Health Administration Medical Toxicology and Environmental Health, Gale Group Health Periodicals Database, *Lancet* Titles, and the *New England Journal of Medicine*.

A total of 4,241 publications were identified from the databases (Fig 2). After the removal of duplicates, the title and abstracts of 3,908 publications were screened for eligibility. After excluding 3,451 publications based on title and abstract screening, 457 full-text publications were assessed for full-text eligibility based on the pre-specified criteria (see Table 1). A total of 339 publications were excluded after full-text screening. Reasons for exclusion were due to the study population (n = 13), intervention (n = 106), outcomes (n = 141), study design (n = 34), geographic scope (n = 44) and language (n = 1). A total of 118 publications were included in the review [28–145] (refer to S1 File for the list of publications excluded after full-text screening).

### Study and intervention characteristics

From a total of 118 publications, a total of 145 single and combination EBIs were identified. These 145 interventions were categorised into the 12 prioritised EBI categories resulting in a total of 225 EBIs (note: number is higher as several interventions involved more than one category of the prioritised EBIs). Results from this review are reported for 225 EBIs (N, number of EBIs).

Most of the 225 EBIs were implemented in the United States (N = 167, 74%) followed by Australia (N = 21, 9%), Canada (N = 13, 6%), United Kingdom (N = 8, 4%), France (N = 10, 4%), Italy (N = 1, <1%) and Spain (N = 5, 2%). No publications were identified for Germany. Of the 12 prioritised EBI categories “rapid access to testing services” was the most frequently implemented EBI (N = 66, 29%) followed by the “role of care navigators” (N = 63, 28%) and “structured follow up” (N = 35, 16%). A variety of study designs was used for the implementation and evaluation of EBIs. The majority of the 225 EBIs were implemented and evaluated using a hybrid study design (N = 94, 42%), followed by quantitative study design (N = 45, 20%), qualitative study design (N = 39, 17%), clinical observational study design (N = 25, 11%) and RCT study design (N = 22, 10%; Table 2). A detailed overview of study and EBI characteristics is provided in S2 Table.

**Table 2:** Overview of EBI study characteristics (N = 225; n = 118) [28–145].

Theme	Prioritised EBI	EBI, N (%)	Distribution of EBIs (N = 225)				
			Country, N	Study Design, N	Implementation Outcome^†^	Theoretical Framework	Effectiveness Outcome^†^
Enabling high accessibility to HIV care services	Regular HIV testing for at-risk groups	11 (5)	Australia: 3 Canada: 1 Spain: 1 USA: 6	RCT: 1 Observational study: 3 Hybrid study: 6 Qualitative study: 1	8	0	4
	Rapid access to testing services	66 (29)	Australia: 13 Canada: 5 France: 8 Italy: 1 Spain: 3 UK: 5 USA: 31	RCT: 5 Observational study: 3 Hybrid study: 36 Qualitative study: 18 Quantitative study: 4	63	2	16
	Rapid ART intervention	2 (1)	USA: 2	Observational study: 2	0	0	2
	One-stop-shop model	2 (1)	USA: 2	Observational study: 2	0	0	2
	Emergency advice service	0 (0)	0	0	0	0	0
Fostering an open and transparent environment	Access to mental health services	7 (3)	USA: 7	RCT: 4 Observational study: 1 Hybrid study: 1 Qualitative study: 1	3	2	6
Creating an optimal care team model	Role of the pharmacist	8 (4)	Australia: 2 Canada: 1 Spain: 1 USA: 4	RCT: 1 Observational study: 1 Hybrid study: 2 Qualitative study: 4	7	0	1
	Role of the care navigators	63 (28)	Australia: 3 Canada: 3 France: 2 UK: 2 USA: 53	RCT: 4 Observational study: 3 Hybrid study: 23 Qualitative study: 12 Quantitative study: 21	56	5	19
Developing a personalized care management model	Individualised plan of care	25 (11)	Canada: 1 USA: 24	RCT: 3 Hybrid study: 10 Quantitative study: 12	21	1	9
Tracking and enabling retention in care	Structured follow-up	35 (16)	Canada: 1 UK: 1 USA: 33	RCT: 4 Observational study: 8 Hybrid study: 13 Qualitative study: 3 Quantitative study: 7	21	3	19
Proactive management of co-infections and co-morbidities	Diagnosis and management of co-infections	2 (1)	Canada: 1 USA: 1	Observational study: 1 Quantitative study: 1	1	0	1
	Diagnosis and management of co-morbidities	4 (2)	USA: 4	Observational study: 1 Hybrid study: 3	3	0	2
	**Total**	**225 (100)**	** **		**183**	**13**	**81**

ART, antiretroviral therapy; EBI, evidence-based intervention; HIV, human immunodeficiency virus; N, number of EBIs identified.

N represents the total number of EBIs included in this review. n represents the number of publications in which these EBIs are evaluated. For study and intervention characteristics, refer to S2 Table.

^†^The sum of EBIs evaluated on implementation and EBIs evaluated on effectiveness do not add up to the total number of EBIs in each category as an EBI was counted in a category if it was at least assessed on any one outcome (i.e., implementation or effectiveness). The categories are not mutually exclusive.

### Evaluation of EBIs

**Fig 3:**
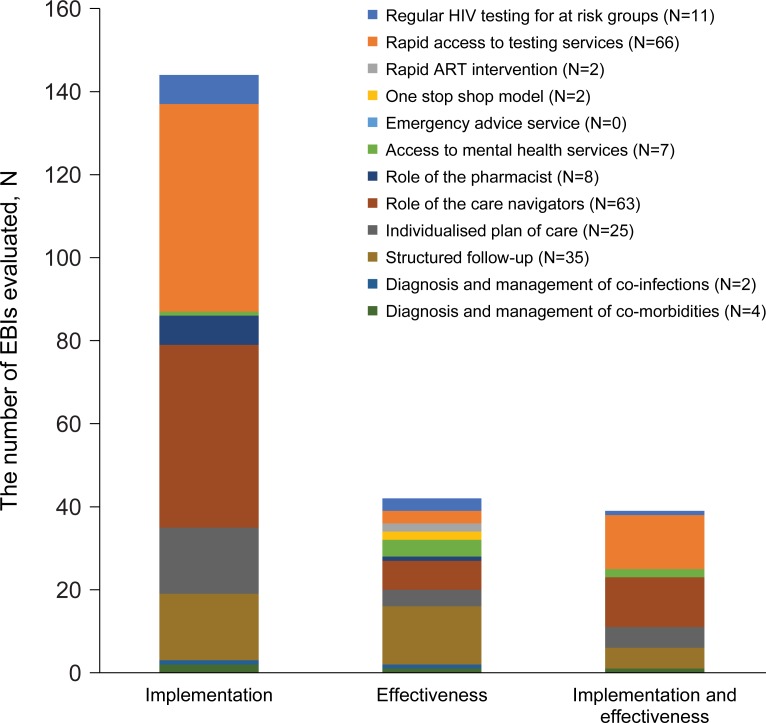
Distribution of EBIs across the evaluation categories (N = 225, n = 118)[28–145]. N represents the total number of EBIs included in this review. n represents the number of publications in which these EBIs are evaluated. ART: antiretroviral therapy; HIV: human immunodeficiency virus.

Fig 3 provides a distribution of the EBIs across the three evaluation categories. Of the 225 EBIs, 144 EBIs were evaluated only on their implementation. “Rapid access to testing services” (N = 50) and “role of care navigators” in HIV care and management (N = 44) were most often evaluated exclusively on their implementation. In total, 42 EBIs were evaluated only on their effectiveness, with the EBIs “structured follow-up” (N = 14) and “role of care navigators” (N = 7) most commonly documented. In addition, 39 EBIs were evaluated on both implementation and effectiveness. Of this latter group, “rapid access to testing services” (N = 13) and “role of care navigators” (N = 12) were most frequently assessed.

Of the 225 EBIs, the majority (N = 183, 81%) were evaluated on their implementation. In addition, a total of 81 EBIs were evaluated on their effectiveness (see Table 2).

#### Implementation outcomes

Of the 183 EBIs where implementation was evaluated, 59 EBIs (32%) involved two implementation outcomes, resulting in a total of 242 documented implementation outcomes. A high level of variability was observed in the definitions of reported implementation outcomes. For example, one study used fidelity to evaluate whether the intervention was delivered as intended. Fidelity was defined by two components, exposure and engagement. The implementers defined exposure as the proportion of text messages sent successfully, and specified engagement with study text messages as the number of months in which a requested response to the study text message was received [30]. In another study, fidelity was used to evaluate the quality and adherence of a trained HIV therapist. The implementers defined quality as the competence of the trained HIV therapist to deliver the intervention and adherence was defined as the % of the session content provided by the HIV therapist being aligned with the study protocol [109].

Across the eight implementation outcomes of interest, acceptability (N = 100) and implementation costs (N = 55) were most often reported. The outcome acceptability was most commonly used for “rapid access to testing services” (N = 48) and “role of care navigators” (N = 22). The assessment of the implementation costs was primarily undertaken in the group of EBIs focused on the “role of care navigators” (N = 24), “individualised plan of care” (N = 12) and “structured follow-up” (N = 10; Table 3). The predominance of implementation cost as an outcome for these EBIs is evident due to the involvement of human resources and their potential impact on healthcare systems.

**Table 3:** Classification of implementation outcomes by EBI (N = 183, n = 93) [28, 30, 31, 35, 36, 40, 42, 43, 45, 46, 48–51, 53–58, 60–64, 66–69, 71, 72, 74, 77–80, 83–94, 96, 97, 99–101, 103, 104, 106–109, 111–114, 116–145].

Type of Intervention	Regular HIV Testing for At Risk Groups (N = 8)	Rapid Access to Testing Services (N = 63)	Rapid ART Intervention (N = 0)	One-Stop Shop Model (N = 0)	Emergency Advice Service (N = 0)	Access to Mental Health Services (N = 3)	Role of the Pharmacist (N = 7)	Role of the Care Navigators (N = 56)	Individualised Plan of Care (N = 21)	Structured Follow-up (n = 21)	Diagnosis and Management of Co-infections (N = 1)	Diagnosis and Management of Comorbidities (N = 3)
Implementation outcome	The proportion of EBIs assessed per implementation outcomes (N)^†^											
Acceptability (N = 100)	7	48	0	0	0	2	5	22	5	8	0	3
Adoption (N = 16)	0	11	0	0	0	0	1	3	1	0	0	0
Appropriateness (N = 3)	0	1	0	0	0	0	1	1	0	0	0	0
Feasibility (N = 35)	1	13	0	0	0	2	0	11	3	5	0	0
Fidelity (N = 16)	0	4	0	0	0	0	0	5	2	4	0	1
Implementation costs (N = 55)	0	8	0	0	0	0	1	24	12	9	1	0
Penetration (N = 2)	0	0	0	0	0	0	0	1	1	0	0	0
Sustainability (N = 15)	2	6	0	0	0	0	0	5	1	0	0	1

N represents the total number of EBIs that are evaluated on implementation. n represents the number of publications in which these EBIs are evaluated.

^†^The numbers reported in the table do not add up to the total number of EBIs evaluated on implementation outcomes (N = 183) as EBIs could be evaluated on more than one implementation outcome.

ART, antiretroviral therapy; EBI, evidence-based intervention; HIV, human immunodeficiency virus.

Of the 242 implementation outcomes reported, 23 implementation outcomes were evaluated by more than one methodological approach, resulting in a total of 265 reported methodologies to evaluate the implementation of the EBIs. The methods reported were classified into three categories: questionnaires, interviews, and frameworks.

**Fig 4:**
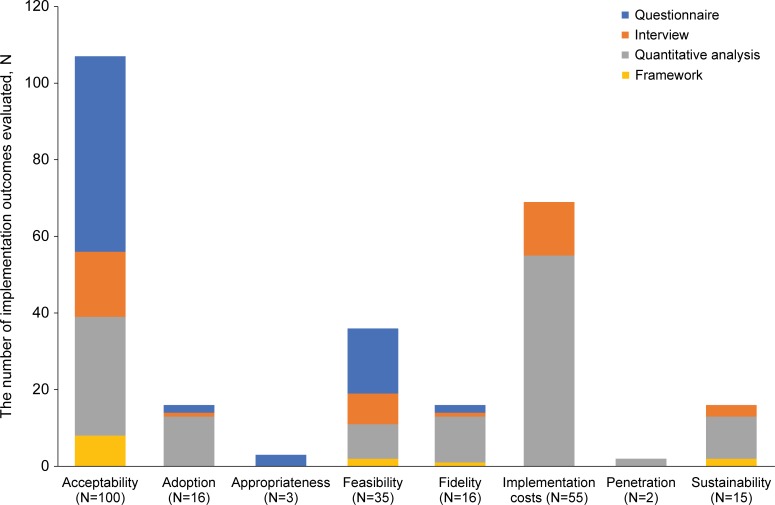
Distribution of methodologies for the evaluation of implementation (N = 242, n = 93) [28, 30, 31, 35, 36, 40, 42, 43, 45, 46, 48–51, 53–58, 60–64, 66–69, 71, 72, 74, 77–80, 83–94, 96, 97, 99–101, 103, 104, 106–109, 111–114, 116–145]. N represents the total number of implementation outcomes reported. n represents the number of publications in which these implementation outcomes are reported. †The numbers reported do not add up to the total number of reported implementation outcomes (N = 242) as multiple methods could be used to evaluate the implementation outcome.

A mix of quantitative and qualitative methods was used to assess the implementation of the EBIs (see Fig 4). For example, in a recent study, the acceptability of an opt-out inpatient HIV screening at an urban teaching hospital was evaluated by using a questionnaire. To determine the acceptability and to describe the predictors of acceptance or refusal of HIV opt-out inpatient testing, surveys were offered to two samples: a) adult patients admitted to the hospital who had been offered an HIV test upon admission over a 3-month period and b) the medical staff of the hospital who offered the HIV tests. The survey consisted of a 5-point Likert-scale and multiple-choice questions [55]. In another study, the acceptability of a mobile health intervention to improve HIV care coordination for PLHIV with co-morbidities was evaluated by applying an interview approach. The first 12 study participants and three peer navigators were asked for their perceptions about the usefulness of the intervention in a one-on-one, in-depth, semi-structured interview [61]. Another example of the use of quantitative methods is a retrospective medical record review of patient-level data in an urban academic medical centre that was used to determine the acceptance rate of HIV testing services and to identify reasons for declining [121].

As presented in Fig 4, amongst all evaluations, only a small number of evaluations (N = 13) involved the use of a theoretical framework. Evaluations involving a theoretical framework were applied to five of the 12 prioritised EBIs, and included: “rapid access to testing services” (N = 2), “access to mental health services” (N = 2), “role of the care navigators” (N = 5), “individualised plan of care” (N = 1) and “structured follow-up” (N = 3). All the EBIs were implemented in the United States, primarily in HIV care clinics (N = 9) and were studies involving fewer than 200 study participants (N = 10). The EBIs were evaluated using the following frameworks: Theory of Reasoned Action and Social Cognitive Theory; ADAPTS framework, Information, Motivation, Behavioural Skills Model; Constant Comparative Method as described by Glaser and Strauss; Grounded theory analysis by Strauss & Corbin; Health Belief Model; Theory of Planned Behaviour and Reasoned Action; Trans-Theoretical Model and Precaution Adoption Process Model [35, 46, 61, 64, 104, 107, 133, 136]. All but one of these frameworks (ADAPTS) originates from disciplines external to implementation science [146] and provides guidance to researchers to study the implementation of an intervention (see Table 4) [136].

**Table 4:** Overview of frameworks used for the evaluation of implementation (N = 13, n = 8) [35, 46, 61, 64, 104, 107, 133, 136].

EBI	Number of EBIs Evaluated by a Framework	Implementation Outcome Assessed	Name of the Framework	**Setting**	**Country of Implementation**	**Population Size, n**
Rapid access to testing services	2	Acceptability Fidelity Sustainability	Theory of Reasoned Action and Social Cognitive Theory ADAPTS Framework	Community Clinic	USA	<200 NR^†^
Access to mental health services	2	Acceptability Feasibility	Information, Motivation, Behavioural Skills Model Multi-stage formative evaluation framework	Clinic (2x)^‡^	USA	<200 (2x)^‡^
Role of the care navigators	5	Feasibility Acceptability Sustainability	Information, Motivation, Behavioural Skills Model (2x) The Constant Comparative Method as described by Glaser and Strauss Grounded theory analysis by Strauss & Corbin Multi-stage formative evaluation (FE) framework	Clinic (4x)^‡^ Hospital	USA	<200 (4x)^‡^ 200–500
Individualised plan of care	1	Acceptability	Combination of the Health Belief Model, the Theory of Planned Behaviour and Reasoned Action, the Trans-Theoretical Model, Precaution Adoption Process Model, and the Information, Motivation, Behavioural Skills meta-theory	Community	USA	<200
Structured follow-up	3	Acceptability Feasibility	Information, Motivation, Behavioural Skills Model Constant Comparative Method as described by Glaser & Strauss Combination of the Health Belief Model, the Theory of Planned Behaviour and Reasoned Action, the Trans-Theoretical Model, Precaution Adoption Process Model, and the Information, Motivation, Behavioural Skills meta-theory	Clinic (2x)^‡^ Community	USA	<200 (2x)^‡^ 200–500

N represents the total number of EBIs that are evaluation with a framework. n represents the number of publications in which these EBIs are evaluated.

^**†**^For this EBI, the number of participants included were not reported.

^‡^ (x) represents the number of times a specific study characteristic has been observed within the EBI category of interest.

ADAPTS, assessment, deliverables, activate, pretraining, training, sustainability; EBI, evidence-based intervention; HIV, human immunodeficiency virus.

#### Effectiveness

In total, 81 EBIs were evaluated on effectiveness outcomes (Table 5). Among the 81 EBIs, 17 EBIs were evaluated for two effectiveness outcomes and one EBI was evaluated for three effectiveness outcomes, resulting in a total of 100 effectiveness outcomes reported overall. Retention in care (N = 42) and linkage to care (N = 41) were more frequently considered for the evaluation of intervention effectiveness compared to medication adherence (N = 17). The effectiveness outcomes reported were consistent with the objectives of the identified EBIs. Evaluation of retention in care was most commonly documented for the following EBIs: “structured follow-up” (N = 14), “role of care navigators” (N = 12) and “individualised plan of care” (N = 6). The EBI “rapid access to testing services” (N = 16), “role of care navigators” (N = 10) and “structured follow-up” (N = 7) were most often evaluated for linkage to care. Medication adherence was evaluated for EBIs that focused on care management, namely: “role of care navigators” (N = 5), “individualised plan of care” (N = 4), “structured follow-up” (N = 4) and “access to mental health services” (N = 3).

**Table 5:** Classification of effectiveness outcomes by EBI category (N = 81, n = 48) [29, 30, 32–35, 37–39, 41, 44, 47, 52, 53, 59, 65, 66, 68–71, 73–76, 81, 82, 89, 90, 93, 95, 97, 98, 100, 102, 105, 109, 110, 115–117, 119, 122, 134, 135, 138, 139, 141].

Type of Intervention	Regular HIV Testing for At-Risk Groups (N = 4)	Rapid Access to Testing Services (N = 16)	Rapid ART Intervention (N = 2)	One Stop Shop Model (N = 2)	Emergency Advice Service (N = 0)	Access to Mental Health Services (N = 6)	Role of the Pharmacist (N = 1)	Role of the Care Navigators (N = 19)	Individualised Plan of Care (N = 9)	Structured Follow-up (N = 19)	Diagnosis and Management of Co-infections (N = 1)	Diagnosis and Management of Comorbidities (N = 2)
Effectiveness outcome	Proportion of EBIs assessed per effectiveness outcomes; N^†^											
Retention to care (N = 42)	2	0	0	1	0	4	1	12	6	14	0	2
Linkage to care (N = 41)	3	16	2	1	0	0	0	10	1	7	1	0
Medication adherence (N = 17)	0	0	0	0	0	3	1	5	4	4	0	0

N represents the total number of EBIs that are evaluation on effectiveness. n represents the number of publications in which these EBIs are evaluated. ART, antiretroviral therapy; HIV, human immunodeficiency virus.

^†^The numbers reported in the table do not add up to the total number of EBIs evaluated on effectiveness outcomes (N = 81) as EBIs could be evaluated on >1 effectiveness outcome.

Significant levels of variation were observed in the evaluation of effectiveness. This was due to the lack of standardised measures and definitions for the measurement of effectiveness. As a result, a wide range of reported measures of effectiveness were observed with too many variations and inconsistencies to report.

## Discussion

With its focus on 12 of the EBIs identified within the Positive Pathways initiative, the findings of this review provide valuable insights into the current state of implementation science in real-world HIV care settings. As such, it provides a valuable context for consideration in the adaptation of EBIs identified in the Positive Pathways initiative, as well as highlighting the progress that remains in maximizing implementation science within HIV to obtain the biggest impact, especially with the 90-90-90 initiative.

In this review, we found 118 publications covering 225 EBIs spanning across the 12 prioritised EBI categories. Of these EBIs, “rapid access to testing services” was most frequently evaluated followed by “role of care navigators” and “structured follow up”. Of these 225 EBIs, 183 were evaluated on implementation. Significant variability was observed in the definitions of reported implementation outcomes. The variability in definitions represents a challenge for implementers to effectively evaluate and understand what EBI works where, how and with whom, as the reported outcomes are not comparable. Consequently, the challenge to bridge gaps in the HIV care continuum remains.

Very few implementation outcomes were being considered for the evaluation of EBIs, which may ultimately limit adaptation of EBIs in the real-world setting. Among the 183 EBIs assessed for implementation outcomes, acceptability and implementation costs were most commonly evaluated, whereas fidelity was rarely reported. This could be attributable to the underlying methods needed to assess these outcomes. Evaluations of acceptability and implementation costs use methods familiar to clinical settings, such as questionnaires and data analysis. In contrast, fidelity measures often require more complex methods, such as an audio and video recording, the development of tailored checklists, and related analyses to assess healthcare professionals’ adherence to study protocols. A mix of quantitative and qualitative methods were used to evaluate the EBIs, however, there was a lack of consistent use of methodologies to evaluate the implementation of EBIs. The variability in reported methodologies suggests that either researchers do not seem to use implementation science approaches, or this variability is perhaps a consequence of the large number of available frameworks. Of the reported evaluations, only 13/183 used a theoretical framework, indicating a knowledge gap in implementation science in HIV. As transferable implementation strategies are required to ensure the consistent use of evidence to change healthcare policy and practice, theoretical frameworks play a key role in implementation science [26, 27]. Only one out of the of the eight frameworks identified in this review provides guidance to researchers to study the implementation of an intervention [136]. The high level of variety in definitions and methodologies used, the disparity in reported implementation outcomes and the minimal use of theoretical frameworks reported in our review suggests that the evaluation of EBIs along the HIV care continuum is not yet aligned with implementation science principles.

Approximately one-third of EBIs were evaluated on effectiveness, most often on linkage and retention in care. Given the critical role that linkage to care plays after HIV diagnosis, it was to be expected that the evaluation of the EBI “rapid access to testing services” was largely measured using linkage to care. In addition, EBIs with a focus on improving care (such as the role of care navigators, individualised care plans and diagnosis and management of co-morbidities) were also evaluated for effectiveness. Substantial levels of variation were observed in the evaluation of effectiveness. Proctor et al. state that implementation outcomes are key intermediate results in relation to clinical effectiveness [23]. Given the inconsistent approaches to assessing implementation, it is not surprising that similar levels of variability were observed with the evaluation of effectiveness, both in terms of definition and methodology used.

Successful implementation of EBIs to support the HIV care continuum in real-world settings, is essential to achieve UNAIDS 90-90-90 targets. However, this review shows evaluations of EBIs in real-world settings, either on implementation or effectiveness outcomes, do not appear to be making optimal use of available implementation science approaches.

These findings corroborate the conclusions of a recent literature review by Hickey et al.[6] which suggested that researchers and implementers continue to face challenges to transfer effective EBIs from one setting to another, or scale up the intervention within the same setting. This could potentially undermine progress toward achieving the UNAIDS 90-90-90 targets. Implementation researchers need to be able to compare and prioritise effective interventions that contribute to achieving optimal health outcomes for PLHIV. It could be argued, that without the consistent use of implementation science, challenges remain to effectively close the gap between evidence and the effective use of EBIs in real-world settings.

We recognise there are limitations to this work. The first phase of the Positive Pathways initiative was to map EBIs in high-income countries and to develop the compendium and self-assessment questionnaire. Before expanding to other geographical regions, we wanted to contextualise the initial findings with a review to understand the extent to which identified EBIs were being evaluated using implementation science. Future reviews of this nature could involve more geographical regions, including low and middle-income countries. Secondly, we restricted our search to a period of five years (2013–2018) and publications written in English only which could potentially limit the generalisability of our findings. Thirdly, we did not conduct all screening activities with two independent reviewers and did not perform a risk of bias analysis for the publications included. As this review aims to obtain a better understanding of the current state of the use of implementation science in HIV and did not aim to evaluate the quality of reported implementation outcomes and methodologies used for evaluation, a detailed data analysis was not included. Therefore, having one researcher conduct screening activities was considered appropriate and a risk of bias analysis not necessary.

Furthermore, this review was not registered in a database for systematic reviews, which may have influenced the level of transparency of this review. However, this review was conducted according to the guidelines in the Cochrane Handbook for Systematic Reviews of Interventions [10] and PRISMA [11] which does minimise the risk of bias in the conduct of this review. In addition, we observed a high level of variability in the definitions used for commonly reported implementation outcomes which required us to interpret where the outcome was best-suited to fit within the taxonomy of the eight Proctor et al. [23] implementation outcomes. In addition, studies using implementation outcomes as a measurement of evaluation were considered eligible for inclusion. However, given the sensitive and non-specific nature of these outcomes, using these outcomes does not mean that implementation science principles were necessarily applied in a study. Given the objective of this review to identify the current state of implementation science in HIV, we qualified a broad range of study designs such as quantitative studies, qualitative studies and hybrid designs, which evaluated either an implementation or effectiveness outcome, or both to this review. It can be argued whether the eligible studies were designed according to accurate implementation science principles, but as both implementation and effectiveness outcomes were of interest for this review, the interrelated link between the two outcomes, and the limited HIV implementation science literature, publications that only reported effectiveness outcomes, without an implementation science focus were also included in this review. For example, an RCT that assessed the retention to care of HIV patients in a real-world setting was considered eligible for inclusion as it reported an effectiveness outcome of interest, even if it did not provide any details about the implementation of the EBI. Adding to this is the inclusion of all hybrid designs that met inclusion criteria for this review. Given that the hybrid methodology is relatively new, these types of study designs remain inconsistently reported by name in the literature. Therefore, studies that reported both on effectiveness and implementation of EBIs were labelled by the reviewers as hybrid design studies using the criteria from Curran et al. [22]. This self-labelling approach may have resulted in misclassification of effectiveness or implementation studies as they may not actually be that type of study design given the variability in interpretation and understanding of these types of studies. This approach is somewhat subjective, and therefore our assessments may be imperfectly reproducible which is not ideal but is reflective of the current state of implementation science in HIV care.

Lastly, this review focused on the taxonomy of implementation outcomes of Proctor et al. [23], as it is widely used and accepted in the field of implementation science for the evaluation of EBIs. The taxonomy provides a classification of implementation outcomes and is therefore often considered in theoretical frameworks that focus on the evaluation of implementation science strategies. Many other theoretical frameworks and models for determining feasible implementation strategies are available. Given the number of theoretical frameworks, it was considered more appropriate to use the taxonomy of Proctor et al. [23] for the search strategy. The inclusion of theoretical frameworks in the search strategy may have restricted the identification of theoretical frameworks and introduce bias. Therefore, in this review only theoretical frameworks that are aligned with Proctor et al. [23] are considered. Overall, the approach used in the current paper likely captures studies that do not meet the strict implementation science criteria. However, we have still captured several implementation studies as evidenced by the proportion of studies citing the use of a hybrid methodology. Regardless, the lack of consistency in labelling a study as implementation research and adhering to proper methodology and reporting in HIV studies in this review remains a large problem. This issue highlights the need for more capacity building in implementation science within HIV research.

## Conclusion

This systematic literature review provides an empirical review of implementation science approaches used to evaluate 12 EBIs in support of the HIV continuum. The learnings from this review highlight the need for a robust implementation science approach to optimise the use of EBIs in HIV care. Variability in how implementation science is applied to HIV, as seen in the ways implementation and effectiveness are evaluated and inconsistency in reporting of measures, methods and outcomes, needs to be addressed if we are to scale up EBIs in support of achieving the UNAIDS 90-90-90 targets. The lack of consistency in application reporting of key implementation science elements found in this study is consistent with the work done in sub-Saharan Africa [147]. Notably, the field of HIV is behind many other fields with respect to utilizing implementation science to improve health outcomes. To successfully scale and replicate EBIs in different settings and contexts there is a need to ensure the use of theoretical frameworks and consistent approaches for the evaluation of implementation outcomes. This will improve understanding of what EBI works where, how and with whom and will bridge the gaps in the HIV care continuum. Importantly, the consistent and accurate utilization and reporting of implementation science components of HIV studies in the future is crucial in our ability to end the epidemic.

## Supporting information

S1 Fig Positive pathways initiative: Development of the compendium of EBIs. **Objective**: The ViiV Healthcare Positive Pathways initiative was established to define best practice and interventions that can effectively close the gaps in the treatment continuum. **Development methodology**: The methodology used to develop the compendium was divided in the three phases. *Phase 1 literature search*: To develop a baseline of the scope and range of activities of current evidence-based practice, a literature search of key published evidence was undertaken from 2010 to 2016. This was a non-systematic literature review using PubMed and Google Scholar as research databases and focusing on geography in the scope of the project. We reviewed a range of articles and journals, from internationally approved guidelines (WHO, IAPAC, CDC, ECDC, NICE and DHHS) for evidence-based practice care and management as well as reviews and observational studies in single centres. An initial list of 66 EBIs was developed from this first phase of the work. *Phase 2 site visits*: In the next phase of the work, we visited eight established centres delivering HIV care to observe current practice and establish the extent and scope of EBIs in use. Centres were identified through the ViiV Healthcare network and selected based on their commitment to UNAIDS 90-90-90 targets and willingness to participate in the initiative including the dissemination of findings through data publication in a relevant peer-reviewed journal. Centres were in Western Europe and North America. Performance data relating to the 90-90-90 targets, were collected and we conducted over 100 interviews across a wide range of stakeholders. An interview guide was created to facilitate structured collection of quantitative and qualitative data on HIV care and management across the care continuum. This included centre and community involvement in HIV awareness and prevention through to disease diagnosis, linkage to and retention in care and clinical management and follow-up. Interview participants were selected by the lead experts within each centre. Selection criteria were based on a participant’s input and level of experience in care and management of HIV patients. We observed HIV care being delivered in a wide range of care settings that included Specialist HIV Centres, Infectious Disease Departments, Sexual Health Clinics and Primary Care Centres, each with specific features. Observations at site visits were cross-referenced against the findings of our secondary literature review to help develop the compendium and HIV care and management assessment questionnaire. *Phase 3 development of compendium*: In the final phase to develop the compendium three advisory boards with 12 experts were held to test and prioritise the key intervention categories and interventions resulting from the first and second phase. Participants included experts from the participating centre, a health economist, patient advocacy group representative and a healthcare systems manager. Within this programme, EBIs were thematically grouped into six categories and prioritised in terms of impact and practicality for implementation leading to the establishment of a final compendium of 21 EBIs. From the compendium of 21 interventions, 12 were prioritized by an expert panel across six key themes of current HIV practice (interventions shaded under each of the six themes). Prioritization was based on a consideration of feasibility/perceived ease for care centres to trial the EBI. These EBIs are expected to be more widely used, investigated and reported. These 12 EBIs from the Positive Pathways initiative were included in the scope of the review.
(DOCX)

S1 Table Medline, Embase, ABI/INFORM, Adis Pharmacoeconomic & Outcomes News, Allied and Complementary Medicine, DH-DATA: Health Administration Medical Toxicology and Environmental Health, Gale Group Health Periodicals Database, *Lancet* Titles, and *New England Journal of Medicine* (via ProQuest). (DOCX)

S2 Table Overview of study and intervention characteristics of publications included in the review (N = 224; n = 118)^†^. (DOCX)

S1 File List of Publications excluded after full-text screening [1–339]. (DOCX)

S2 File Cox PRISMA checklist. (DOC)
