# Supplementary material for: A need for implementation science to optimise the use of evidence-based interventions in HIV care: A systematic literature review
Source: PLoS One. 2019 Aug 19;14(8):e0220060. doi: 10.1371/journal.pone.0220060 (PMC6699703; doi:10.1371/journal.pone.0220060)
Supplement: S1 Table — (DOCX) [file pone.0220060.s002.docx]

S1 Table. Medline, Embase, ABI/INFORM, Adis Pharmacoeconomic & Outcomes News, Allied and Complementary Medicine, DH-DATA: Health Administration Medical Toxicology and Environmental Health, Gale Group Health Periodicals Database, *Lancet* Titles, and *New England Journal of Medicine* (via ProQuest).

| **Topic** | **Set#** | **Search Phrase/Term** | **Results** |
| --- | --- | --- | --- |
| **Indication: HIV**  Search terms of original review used | S1 | TI,AB,IF(HIV) OR TI,AB,IF("human immunodeficiency virus") | 966238* |
|  | S2 | TI,AB,IF(AIDS) OR TI,AB,IF("acquired immune deficiency syndrome") | 1530194* |
|  | S3 | MJEMB.EXACT("Human immunodeficiency virus") OR MJEMB.EXACT("acquired immune deficiency syndrome") | 190736* |
|  | S4 | MJMESH.EXACT("HIV") OR MJMESH.EXACT("Acquired Immunodeficiency Syndrome") | 70521* |
|  | S5 | S1 OR S2 OR S3 OR S4 | 2171625* |
| **12 prioritised interventions**  Search terms of original review 2 | S6 | TI,AB,IF(accessibility NEAR/2 care) OR TI,AB,IF((open OR transparent) NEAR/2 care) OR TI,AB,IF(optimal NEAR/3 team) OR TI,AB,IF(retent* NEAR/2 care) OR TI,AB,IF(care NEAR/1 cascade) OR TI,AB,IF(90-90-90) | 33734* |
|  | S7 | ((regular NEAR/5 test*) OR (regular NEAR/5 exam*)) AND (high*risk) | 58° |
|  | S8 | (rapid NEAR/5 test*) OR (quick NEAR/5 test*) OR (express NEAR/5 test*) OR (access* NEAR/2 "HIV gadget") | 162382* |
|  | S9 | (rapid NEAR/5 "antiretroviral therapy") OR (rapid NEAR/5 ART) OR (quick NEAR/5 "antiretroviral therapy") OR (quick NEAR/5 ART) OR (immediate NEAR/5 "antiretroviral therapy") OR (fast NEAR/5 "antiretroviral therapy") OR (fast NEAR/5 ART) | 15515* |
|  | S10 | (one*stop*shop) OR (integrat* NEAR manag*) | 636036* |
|  | S11 | (emergent NEAR/10 advice) OR (emergency NEAR/10 advice) OR (emergent NEAR/10 consult*) OR hotline OR (Walk*in NEAR/2 clinic*) | 171760* |
|  | S12 | TI,AB("mental health") | 518095* |
|  | S13 | TI,AB(pharmacist*) | 157361* |
|  | S14 | (care NEAR/2 navigator*) OR (peer NEAR/2 navigator*) OR (care NEAR/2 navigation) OR (peer NEAR/2 navigation) | 3137° |
|  | S15 | (individualized NEAR/2 care) OR (individual NEAR/2 care) OR (personalized NEAR/2 care) | 126593* |
|  | S16 | (structured NEAR/2 follow*up) OR (schedule* NEAR/2 follow*up) | 343° |
|  | S17 | ((diagnos* OR manag*) NEAR/2 co*morbidit*) OR ((diagnos* OR manag*) NEAR/2 co*infect*) | 20668* |
|  | S18 | S6 OR S7 OR S8 OR S9 OR S10 OR S11 OR S12 OR S13 OR S14 OR S15 OR S16 OR S17 | 1818873* |
| **Implementation science**  Search terms of original review 1, Proctor et al.[23] and KT Canada[147] terms have been used | S19 | TI,AB,IF("implementation science") | 1686° |
|  | S20 | TI,AB,IF(delivery NEAR/2 science) | 2053° |
|  | S21 | TI,AB,IF(dissemination NEAR/2 science) | 536° |
|  | S22 | TI,AB,IF(innovation NEAR/2 implementation) | 2085° |
|  | S23 | TI,AB,IF(evidence NEAR/2 informed decision making) | 616° |
|  | S24 | TI,AB,IF(knowledge NEAR/5 translation) | 8317* |
|  | S25 | TI,AB,IF(implementation NEAR/5 research) | 23089* |
|  | S26 | TI,AB,IF(gap NEAR/2 evidence) | 6665* |
|  | S27 | TI,AB,IF(gap NEAR/2 knowledge) | 38220* |
|  | S28 | TI,AB,IF(gap NEAR/2 practice) | 5847* |
|  | S29 | TI,AB,IF("optimize use" NEAR/2 intervention) | 4° |
|  | S30 | TI,AB,IF("optimize use" NEAR/2 initiative) | 2° |
|  | S31 | TI,AB,IF("optimize use" NEAR/2 program) | 1° |
|  | S32 | TI,AB,IF("real world" NEAR/2 intervention) | 483° |
|  | S33 | TI,AB,IF(strategy NEAR/2 adheren*) | 2030° |
|  | S34 | TI,AB,IF(retent* NEAR/2 strategy) | 5101* |
|  | S35 | TI,AB,IF(optimiz* NEAR/2 biological) | 1915° |
|  | S36 | TI,AB,IF(90-90-90) | 561° |
|  | S37 | TI,AB,IF(("evidence-based" OR "evidence based") NEAR/2 medicine) | 64797* |
|  | S38 | TI,AB,IF(clinical NEAR/2 medicine) | 101315* |
|  | S39 | TI,AB,IF("health services") | 864129* |
|  | S40 | TI,AB,IF(randomized controlled trial) | 787769* |
|  | S41 | TI,AB,IF(evaluation NEAR/5 research) | 83947* |
|  | S42 | EMB.EXACT("randomized controlled trial") | 542464* |
|  | S43 | MESH.EXACT("Randomized Controlled Trials as Topic") | 117288* |
|  | S44 | MESH.EXACT("Evidence-Based Medicine") | 68605* |
|  | S45 | EMB.EXACT("evidence based medicine") | 100819* |
|  | S46 | EMB.EXACT("clinical medicine") | 9983* |
|  | S47 | MESH.EXACT("Clinical Medicine") | 5303* |
|  | S48 | EMB.EXACT("evaluation research") | 2236° |
|  | S49 | MESH.EXACT("Evaluation Studies as Topic") | 122056* |
|  | S50 | TI,AB,IF(accept* NEAR/4 (program* OR intervention or initiative)) OR TI,AB,IF(satisf* NEAR/4 (program* OR intervention OR initiative)) OR EMB.EXACT("program acceptability") OR EMB.EXACT("patient satisfaction") OR MESH.EXACT("Patient Satisfaction") OR MESH.EXACT("Patient Acceptance of Health Care") | 283539* |
|  | S51 | TI,AB,IF(adopt* NEAR/4 (program* OR intervention OR initiative)) OR TI,AB,IF(uptake NEAR/4 (program* OR intervention OR initiative)) OR TI,AB,IF(utiliz* NEAR/4 (program* OR intervention OR initiative)) | 72664* |
|  | S52 | TI,AB,IF(appropriate* NEAR/4 (program* OR intervention OR initiative)) OR TI,AB,IF((compatibility OR compatible) NEAR/4 (program* OR intervention OR initiative)) OR TI,AB,IF((suitability OR suitable) NEAR/4 (program* OR intervention OR initiative)) OR TI,AB,IF((practicability OR practicable) NEAR/4 (program* OR intervention OR initiative)) OR EMB.EXACT("program appropriateness") | 66610* |
|  | S53 | TI,AB,IF((feasible OR feasibility) NEAR/4 (program* OR intervention OR initiative)) OR EMB.EXACT("program feasibility") | 24907* |
|  | S54 | TI,AB,IF(fidelity NEAR/4 (program* OR intervention OR initiative)) OR TI,AB,IF(integrity NEAR/4 (program* OR intervention OR initiative)) OR TI,AB,IF(quality NEAR/4 "program delivery") | 9860* |
|  | S55 | TI,AB,IF(cost NEAR/4 (program* OR intervention OR initiative)) AND TI,AB,IF(implementation) | 9237* |
|  | S56 | TI,AB,IF(penetrat* NEAR/4 (program* OR intervention OR initiative)) | 1423° |
|  | S57 | TI,AB,IF(sustainab* NEAR/4 (program* OR intervention OR initiative)) OR TI,AB,IF(maintenance NEAR/4 (program* OR intervention OR initiative)) OR TI,AB,IF(incorporat* NEAR/4 (program* OR intervention OR initiative)) OR TI,AB,IF(durability NEAR/4 (program* OR intervention OR initiative)) OR TI,AB,IF(institutionaliz* NEAR/4 (program* OR intervention OR initiative)) OR TI,AB,IF(routiniz* NEAR/4 (program* OR intervention OR initiative)) OR EMB.EXACT("program sustainability") | 86536* |
|  | S58 | S19 OR S20 OR S21 OR S22 OR S23 OR S24 OR S25 OR S26 OR S27 OR S28 OR S29 OR S30 OR S31 OR S32 OR S33 OR S34 OR S35 OR S36 OR S37 OR S38 OR S39 OR S40 OR S41 OR S42 OR S43 OR S44 OR S45 OR S46 OR S47 OR S48 OR S49 OR S50 OR S51 OR S52 OR S53 OR S54 OR S55 OR S56 OR S57 | 2861618* |
| **Country limit**  Limit is taken directly from original review | S59 | "United States" OR "USA " OR "US " OR "U.S." OR America OR American | 110118832* |
|  | S60 | Europe OR European | 13003106* |
|  | S61 | "United Kingdom" OR "UK " OR "Great-Britain" OR "Great Britain" OR British OR England OR English OR Wales OR Welsh OR Scotland OR Scottish OR Ireland OR Irish | 159024094* |
|  | S62 | France OR French | 7359336* |
|  | S63 | Germany OR German | 10290728* |
|  | S64 | Italy OR Italian | 4660965* |
|  | S65 | Spain OR Spanish | 4860364* |
|  | S66 | Canada OR Canadian | 11441710* |
|  | S67 | Australia OR Australian | 9192005* |
|  | S68 | S59 OR S60 OR S61 OR S62 OR S63 OR S64 OR S65 OR S66 OR S67 | 164425158* |
| **Language limit**  Limit is taken directly from original review | S69 | la.exact(ENG) | 154394040* |
| **Time limit**  Limit is shorter than original review (5 instead of 10 years). This limit identifies relevant implementation interventions, strategies and outcomes | S70 | PD(>20130101) | 56026847* |
| **Total** | S71 | S5 AND S18 AND S58 AND S68 AND S69 AND S70 | 3863° |

Search date: 29 March 2018. Please refer to the manuscript for a list of the references used.
