# Supplementary material for: A need for implementation science to optimise the use of evidence-based interventions in HIV care: A systematic literature review
Source: PLoS One. 2019 Aug 19;14(8):e0220060. doi: 10.1371/journal.pone.0220060 (PMC6699703; doi:10.1371/journal.pone.0220060)
Supplement: S2 Table — (DOCX) [file pone.0220060.s003.docx]

S2 Table. Overview of Study and Intervention Characteristics of Publications Included in the Review (N=224; n=118).^†^

| **General Information** | | **Intervention Details** | | | | **Implementation Outcomes** | | | **Effectiveness Outcomes** |
| --- | --- | --- | --- | --- | --- | --- | --- | --- | --- |
| **First Author, Year** | **Study Design** | **Type of Intervention** | **Setting** | **Country** | **Population Size** | **Implementation Parameter** | **Methodology** | **Framework** | **Measurement** |
| Addison, 2018 [28] | Hybrid study | Role of care navigators | Clinic | USA | 200-500 | Adoption; Penetration | Quantitative analysis; Quantitative analysis | NR | NR |
|  |  | Structured follow up | Clinic | USA | >1000 | Feasibility; Fidelity | Quantitative analysis; Quantitative analysis | NR | NR |
|  |  | Individualized plan of care | Clinic | USA | <200 | Adoption; Penetration | Quantitative analysis; Quantitative analysis | NR | Linkage to care |
| Bentsianov, 2018 [29] | Observational study | Rapid access to testing services; Rapid ART intervention | Community | USA | >1000 | NR | NR | NR | Linkage to care |
| Christopoulos, 2018 [30] | Hybrid study | Structured follow up | Clinic; Community | USA | 200-500 | Fidelity; Acceptability | Quantitative analysis; Questionnaire | NR | Retention to care |
| Demorat, 2018 [31] | RCT | Rapid access to testing services | GP | France | 200-500 | Acceptability; Feasibility | Quantitative analysis; Quantitative analysis | NR | NR |
|  |  | Rapid access to testing services | GP | France | <200 | Acceptability; Feasibility | Quantitative analysis; Quantitative analysis | NR | NR |
|  |  | Rapid access to testing services | GP | France | <200 | Acceptability; Feasibility | Quantitative analysis; Quantitative analysis | NR | NR |
| Menza, 2018 [32] | Hybrid study | Structured follow up | NR | USA | <200 | NR | NR | NR | Retention to care; Medication adherence |
| Saag, 2018 [33] | Observational study | Access to mental health services; Diagnosis & management of co-morbidities | Clinic | USA | 500-1000 | NR | NR | NR | Retention to care |
| Shacham, 2018 [34] | Hybrid study | Individualized plan of care; Role of care navigators | Clinic | USA | 200-500 | NR | NR | NR | Retention to care |
| Smith, 2018 [35] | RCT | Role of care navigators; Access to mental health services | Clinic | USA | <200 | Feasibility; Acceptability | Questionnaire; Quantitative analysis; Framework | Information, Motivation, Behavioral Skills (sIMB) Model | Retention to care |
| Yee, 2018 [36] | Observational study | Regular HIV testing for at risk groups | Hospital | USA | >1000 | Sustainability | Quantitative analysis | NR | NR |
| Bean, 2017 [37] | Observational study | Structured follow up; Role of care navigators | Clinic; Other | USA | <200 | NR | NR | NR | Retention to care |
| Bouris, 2017 [38] | RCT | Regular HIV testing for at risk groups | Clinic; Community | USA | <200 | NR | NR | NR | Retention to care |
| Castel, 2017 [39] | Observational study | Rapid access to testing services | Community | USA | >1000 | NR | NR | NR | Linkage to care |
| Coleman, 2017 [40] | Hybrid study | Structured follow up; Role of care navigators | Other | USA | <200 | Acceptability; Feasibility | Quantitative analysis; Quantitative analysis | NR | NR |
| Cunningham, 2017 [41] | RCT | Role of care navigators | Jail; Community | USA | <200 | NR | NR | NR | Linkage to care; Retention to care; Medication adherence |
| Davies, 2017 [42] | Hybrid study | Rapid access to testing services | Clinic | Australia | 200-500 | Acceptability; Adoption | Questionnaire; Questionnaire | NR | NR |
| Dong, 2017 [43] | Observational study | Role of the pharmacist | Other; Jail | USA | <200 | Adoption | Quantitative analysis | NR | NR |
| Gómez, 2017 [44] | Observational study | Structured follow up; Role of care navigators | Community; Jail | USA | 500-1000 | NR | NR | NR | Linkage to care |
| Hsieh, 2017 [45] | Hybrid study | Rapid access to testing services | Hospital; Other | USA | 200-500 | Acceptability | Quantitative analysis | NR | NR |
| Johnson, 2017 [46] | Hybrid study | Rapid access to testing services | Other | USA | <200 | Acceptability; Fidelity | Questionnaire; Framework | Theory of Reasoned Action and Social Cognitive Theory | NR |
| Kutnick, 2017 [47] | Hybrid study | Structured follow up; Role of care navigators | Community | USA | <200 | NR | NR | NR | Linkage to care; Retention to care |
| Leblanc, 2017 [48] | RCT | Rapid access to testing services | Hospital | France | >1000 | Adoption; Acceptability | Questionnaire; Quantitative analysis | NR | NR |
| Leblanc, 2017 [49] | Hybrid study | Rapid access to testing services; NR | Hospital | France | >1000 | Implementation costs; Acceptability | Quantitative analysis; Questionnaire | NR | NR |
| Mackie, 2017 [50] | Hybrid study | Role of the pharmacist | GP | Australia | <200 | Acceptability | Questionnaire | NR | NR |
| Maulsby, 2017 [51] | Quantitative study | Role of care navigators; Individualized plan of care | Community | USA | <200 | Implementation costs | Quantitative analysis; Interview | NR | NR |
|  |  | Role of care navigators; Individualized plan of care | Community | USA | <200 | Implementation costs | Quantitative analysis; Interview | NR | NR |
|  |  | Role of care navigators; Individualized plan of care | Community | USA | <200 | Implementation costs | Quantitative analysis; Interview | NR | NR |
|  |  | Role of care navigators; Individualized plan of care | Community | USA | >1000 | Implementation costs | Quantitative analysis; Interview | NR | NR |
|  |  | Role of care navigators; Individualized plan of care | Community | USA | <200 | Implementation costs | Quantitative analysis; Interview | NR | NR |
|  |  | Role of care navigators; Individualized plan of care | Community | USA | <200 | Implementation costs | Quantitative analysis; Interview | NR | NR |
|  |  | Role of care navigators; Individualized plan of care | Community | USA | <200 | Implementation costs | Quantitative analysis; Interview | NR | NR |
| Metcalfe, 2017 [52] | Observational study | Structured follow up | Hospital | UK | <200 | NR | NR | NR | Linkage to care |
| Moitra, 2017 [53] | RCT | Role of care navigators; NR | Hospital; clinic | USA | <200 | Acceptability; Feasibility | Quantitative analysis; Questionnaire | NR | Retention to care |
| Mutch, 2017 [54] | Hybrid study | Rapid access to testing services | Community | Australia | >1000 | Acceptability | Questionnaire; Other | NR | NR |
| Osorio, 2017 [55] | Hybrid study | Rapid access to testing services | Hospital | USA | >1000 | Acceptability; Adoption | Questionnaire; Quantitative analysis | NR | NR |
|  |  | Rapid access to testing services | Hospital | USA | >1000 | Acceptability; Adoption | Questionnaire; Quantitative analysis | NR | NR |
|  |  | Rapid access to testing services | Hospital | USA | >1000 | Acceptability; Adoption | Questionnaire; Quantitative analysis | NR | NR |
| Patel, 2017 [56] | RCT | Rapid access to testing services | Hospital | USA | <200 | Acceptability | Quantitative analysis | NR | NR |
| Ryan, 2017 [57] | Qualitative study | Rapid access to testing services | Clinic | Australia | >1000 | Acceptability | Quantitative analysis; Interview | NR | NR |
| Senn, 2017 [58] | Hybrid study | Role of care navigators | Clinic; Other | USA | <200 | Feasibility; | Interview; Questionnaire | NR | NR |
| Tesoriero, 2017 [59] | Observational study | Structured follow up; Role of care navigators | NR | USA | 200-500 | NR | NR | NR | Linkage to care; Retention to care |
| Uccella, 2017 [60] | Hybrid study | Rapid access to testing services | Clinic | Italy | 500-1000 | Acceptability | Questionnaire | NR | NR |
| Westergaard, 2017 [61] | Qualitative study | Role of care navigators; Structured follow up | Clinic; Other | USA | <200 | Acceptability; NR | Interview; Framework | Information, Motivation, Behavioral Skills (sIMB) Model | NR |
| Brondani, 2016 [63] | Qualitative study | Rapid access to testing services; NR | Clinic | Canada | >1000 | Acceptability | Questionnaire | NR | NR |
| Centre for Epidemiological Studies on HIV/STI in Catalonia (CEEISCAT), 2016 [63] | Hybrid study | Regular HIV testing for at risk groups; Rapid access to testing services | Hospital; Community | Spain | 200-500 | Acceptability; Feasibility | Questionnaire; NR | NR | NR |
| Drummond, 2016 [64] | Qualitative study | Access to mental health services; Role of care navigators | Clinic | USA | <200 | Acceptability | Framework; Interview | Multi-stage formative evaluation (FE) framework | NR |
| Gardner,2016 [65] | RCT | Structured follow up; Access to mental health services | Clinic | USA | >1000 | NR | NR | NR | Retention to care |
| Giordano, 2016 [66] | Hybrid study | Role of care navigators | Hospital; Clinic | USA | 200-500 | Fidelity | Interview | NR | Retention to care; Medication adherence |
| Jain, 2016 [67] | Quantitative study | Role of care navigators; Individualized plan of care | Jail; Other | USA | NR | Implementation costs | Quantitative analysis | NR | NR |
|  |  | Role of care navigators; Individualized plan of care | NR | USA | NR | Implementation costs | Quantitative analysis | NR | NR |
|  |  | Role of care navigators; Structured follow up | NR | USA | NR | Implementation costs | Quantitative analysis | NR | NR |
|  |  | Role of care navigators; Individualized plan of care | NR | USA | NR | Implementation costs | Quantitative analysis | NR | NR |
|  |  | Role of care navigators; Structured follow up | Clinic | USA | NR | Implementation costs | Quantitative analysis | NR | NR |
|  |  | Role of care navigators; Structured follow up | NR | USA | NR | Implementation costs | Quantitative analysis | NR | NR |
|  |  | Role of care navigators; Structured follow up | Clinic | USA | NR | Implementation costs | Quantitative analysis | NR | NR |
|  |  | Role of care navigators; Individualized plan of care | Community | USA | NR | Implementation costs | Quantitative analysis | NR | NR |
|  |  | Role of care navigators; Structured follow up | Community | USA | NR | Implementation costs | Quantitative analysis | NR | NR |
|  |  | Role of care navigators; Structured follow up | Jail; Other | USA | NR | Implementation costs | Quantitative analysis | NR | NR |
|  |  | Role of care navigators; Structured follow up | Clinic | USA | NR | Implementation costs | Quantitative analysis | NR | NR |
|  |  | Role of care navigators; Individualized plan of care | NR | USA | NR | Implementation costs | Quantitative analysis | NR | NR |
| Jeffries, 2016 [68] | Hybrid study | Structured follow up | Community | USA | <200 | Acceptability | Questionnaire | NR | Retention to care |
| Kenya, 2016 [69] | Hybrid study | Role of care navigators; Rapid access to testing services | Community | USA | <200 | Acceptability; Fidelity | Questionnaire; Quantitative analysis | NR | Linkage to care |
| Lucas, 2016 [70] | Observational study | Rapid access to testing services; Rapid ART intervention | Jail | USA | >1000 | NR | NR | NR | Linkage to care |
| Metsch, 2016 [71] | Hybrid study | Role of care navigators; Structured follow up | Hospital; Community | USA | 200-500 | Fidelity | Quantitative analysis | NR | Linkage to care |
|  |  | Role of care navigators; Structured follow up | Hospital; Community | USA | 200-500 | Fidelity | Quantitative analysis | NR | Linkage to care |
| Préau, 2016 [72] | Hybrid study | Rapid access to testing services; Role of care navigators | CBO | France | 200-500 | Acceptability | Quantitative analysis | NR | NR |
| Safren, 2016 [73] | RCT | Access to mental health services; Individualized plan of care | Clinic | USA | 200-500 | NR | NR | NR | Medication adherence |
| Stenstrom, 2016 [74] | Hybrid study | Rapid access to testing services | Hospital | Canada | >1000 | Acceptability | Questionnaire | NR | Linkage to care |
| Xiao, 2016 [75] | Observational study | One stop shop model; Structured follow up | Clinic; Community | USA | <200 | NR | NR | NR | Retention to care |
| Bove, 2015 [76] | Observational study | Structured follow up | Clinic | USA | 500-1000 | NR | NR | NR | Retention to care |
| Chan, 2015 [77] | Hybrid study | Rapid access to testing services | Clinic; Other | Australia | >1000 | Acceptability | Questionnaire | NR | NR |
| Conway, 2015 [78] | Qualitative study | Rapid access to testing services; Role of care navigators | Clinic | Australia | <200 | Acceptability; Feasibility | Questionnaire; Questionnaire | NR | NR |
| Conway, 2015 [79] | Qualitative study | Rapid access to testing services; Role of care navigators | Clinic | Australia | >1000 | Acceptability | Questionnaire | NR | NR |
| Fernández-Balbuena, 2015 [80] | Qualitative study | Rapid access to testing services; Role of the pharmacist | Pharmacy | Spain | >1000 | Acceptability; Implementation costs | Questionnaire; Quantitative analysis | NR | NR |
| Flash,2015 [81] | Observational study | Regular HIV testing for at risk groups | Hospital | USA | >1000 | NR | NR | NR | Linkage to care; Retention to care |
| Hakobyan, 2015 [82] | Observational study | Regular HIV testing for at risk groups; Diagnosis & management of co-infections | Community; Clinic | Canada | >1000 | NR | NR | NR | Linkage to care |
| Hood, 2015 [83] | Hybrid study | Rapid access to testing services; Role of care navigators | CBO | USA | 500-1000 | Sustainability; Acceptability | Quantitative analysis; Questionnaire | NR | NR |
| Hale, 2015 [84] | Qualitative study | Role of the pharmacist | Hospital; Clinic | Australia | 200-500 | Appropriateness | Questionnaire | NR | NR |
| Katz, 2015  [85] | Hybrid study | Rapid access to testing services | Other | USA | 200-500 | Adoption; Feasibility | Quantitative analysis; Quantitative analysis | NR | NR |
| Kielly, 2015 [86] | Qualitative study | Role of the pharmacist; Role of care navigators | Clinic | Canada | <200 | Acceptability | Questionnaire | NR | NR |
| Kim, 2015 [87] | Hybrid study | Role of care navigators; Structured follow up | Community | USA | >1000 | Implementation costs; | Quantitative analysis | NR | NR |
| Knapp, 2015 [88] | Quantitative study | Rapid access to testing services; Role of care navigators | Hospital | USA | >1000 | Implementation costs; Sustainability | Quantitative analysis; Quantitative analysis | NR | NR |
| Knight, 2015 [89] | Hybrid study | Rapid access to testing services | Clinic | Australia | 500-1000 | Adoption | Quantitative analysis | NR | Linkage to care |
|  |  | Rapid access to testing services | CBO | Australia | 500-1000 | Adoption | Quantitative analysis | NR | Linkage to care |
|  |  | Rapid access to testing services | Other | Australia | 200-500 | Adoption | Quantitative analysis | NR | Linkage to care |
| Leber, 2015 [90] | Hybrid study | Rapid access to testing services | GP | UK | >1000 | Acceptability | Quantitative analysis | NR | Linkage to care |
| Lecher, 2015 [91] | Quantitative study | Rapid access to testing services; Role of care navigators | Pharmacy; Clinic | USA | 500-1000 | Implementation costs | Quantitative analysis | NR | NR |
| Lessard, 2015 [92] | Qualitative study | Rapid access to testing services | Clinic | Canada | <200 | Acceptability | Interview | NR | NR |
| MacGowan, 2015 [93] | Hybrid study | Individualized plan of care; Role of care navigators | Jail; Other | USA | <200 | Fidelity | Quantitative analysis | NR | Retention to care; Medication adherence |
| Maturo, 2015 [94] | Hybrid study | Individualized plan of care; Structured follow up | Clinic | USA | <200 | Feasibility | Questionnaire | NR | NR |
| Maulsby, 2015 [95] | Observational study | One stop shop model; Structured follow up | Community; Clinic | USA | >1000 | NR | NR | NR | Linkage to care |
| Metsch,2015  [96] | Hybrid study | Diagnosis & management of co-morbidities; Individualized plan of care | Clinic | USA | 500-1000 | Sustainability; Acceptability | Interview; Interview | NR | NR |
| Moitra, 2015 [97] | Hybrid study | Role of care navigators; Individualized plan of care | Clinic | USA | <200 | Acceptability; Feasibility | Questionnaire; Questionnaire | NR | Retention to care |
| Pence, 2015 [98] | RCT | Access to mental health services; Role of care navigators | Clinic | USA | 200-500 | NR | NR | NR | Medication adherence; Retention to care |
| Poirier, 2015 [99] | Hybrid study | Rapid access to testing services; Role of care navigators | GP | France | <200 | Appropriateness; Adoption | Questionnaire; Questionnaire | NR | NR |
| Prekker, 2015 [100] | Hybrid study | Rapid access to testing services | Hospital | USA | >1000 | Acceptability; Feasibility | Questionnaire; Questionnaire | NR | Linkage to care |
| Schackman, 2015 [101] | Quantitative study | Rapid access to testing services; Diagnosis & management of co-infections | Community | USA | >1000 | Implementation costs | Quantitative analysis | NR | NR |
| Schafer, 2015 [102] | RCT | Structured follow up; Role of the pharmacist | Clinic | USA | <200 | NR | NR | NR | Retention to care; Medication adherence |
| Shrestha, 2015 [103] | Hybrid study | Role of care navigators; Structured follow up | Clinic | USA | >1000 | Implementation costs | Quantitative analysis | NR | NR |
| Swendeman, 2015 [104] | Hybrid study | Individualized plan of care; Structured follow up | Community | USA | <200 | Acceptability | Framework; Interview | Social Cognitive Theory, the Health Belief Model, the Theory of Planned Behavior and Reasoned Action, the Trans-Theoretical Model, Precaution Adoption Process Model, and the Information, Motivation, Behavioral Skillsmeta-theory. | NR |
| Terzian, 2015 [105] | Observational study | Structured follow up | Community | USA | >1000 | NR | NR | NR | Retention to care |
| Bamford, 2014 [106] | Hybrid study | Rapid access to testing services | Hospital | USA | >1000 | Acceptability | Questionnaire | NR | NR |
| Drainoni, 2014 [107] | Qualitative study | Structured follow up; Role of care navigators | Clinic | USA | 200-500 | Feasibility; Acceptability | Framework; Framework | The Constant Comparative Method as described by Glaser and Strauss | NR |
| Eggman, 2014 [108] | Quantitative study | Rapid access to testing services | Clinic | USA | NR | Implementation costs | Quantitative analysis | NR | NR |
| Esposito-Smythers, 2014 [109] | Hybrid study | Individualized plan of care; Diagnosis & management of co-morbidities | Clinic | USA | <200 | Fidelity; Acceptability | Questionnaire; Questionnaire | NR | Retention to care |
| Gardner, 2014 [110] | RCT | Individualized plan of care; Structured follow up | Clinic | USA | >1000 | NR | NR | NR | Retention to care |
| Hooshyar, 2014 [111] | Qualitative study | Rapid access to testing services | Community | USA | 500-1000 | Acceptability; Fidelity | Quantitative analysis; Quantitative analysis | NR | NR |
| Kasting, 2014 [112] | Hybrid study | Regular HIV testing for at risk groups | NR | USA | >1000 | Acceptability | Questionnaire | NR | NR |
| Knapp, 2014 [113] | Qualitative study | Rapid access to testing services; Role of care navigators | Clinic | USA | <200 | Sustainability; Feasibility | Quantitative analysis; Interview | NR | NR |
| Knapp, 2014 [114] | Qualitative study | Rapid access to testing services; Role of care navigators | Hospital | USA | <200 | Sustainability; Feasibility | Quantitative analysis; Interview | NR | NR |
| Konkle-Parker, 2014 [115] | RCT | Structured follow up; Individualized plan of care | Clinic | USA | <200 | NR | NR | NR | Retention to care; Medication adherence |
| Magidson, 2014 [116] | Hybrid study | Access to mental health services; Individualized plan of care | Community | USA | <200 | Feasibility | Questionnaire | NR | Medication adherence |
| Marlin, 2014 [117] | Qualitative study | Rapid access to testing services | CBO | USA | <200 | Acceptability | Interview | NR | Linkage to care |
| Merchant, 2014 [118] | Hybrid study | Rapid access to testing services; Role of care navigators | Hospital | USA | 200-500 | Adoption; Acceptability | Quantitative analysis; Questionnaire | NR | NR |
| O'Connor, 2014 [119] | Hybrid study | Rapid access to testing services; Role of care navigators | Hospital | UK | >1000 | Acceptability | Quantitative analysis | NR | Linkage to care |
| Rosales-Statkus, 2014 [120] | Qualitative study | Rapid access to testing services | Other | Spain | 200-500 | Feasibility | Questionnaire | NR | NR |
| Schechter-Perkins, 2014 [121] | Hybrid study | Rapid access to testing services; | Hospital | USA | >1000 | Acceptability | Quantitative analysis | NR | NR |
| Smillie, 2014 [122] | Hybrid study | Structured follow up; Role of care navigators | Clinic | Canada | <200 | Feasibility; Acceptability | Interview; Interview | NR | Medication adherence; Retention to care |
| Vachirasudlekha, 2014 [123] | Qualitative study | Role of care navigators; Structured follow up; role of the pharmacist | Clinic | USA | <200 | Acceptability | Questionnaire | NR | NR |
| Yang, 2014 [124] | Hybrid study | Regular HIV testing for at risk groups; Rapid access to testing services | Clinic; Community | Australia | 500-1000 | Acceptability | Questionnaire | NR | NR |
| Young, 2014 [125] | Qualitative study | Rapid access to testing services | Other | USA | <200 | Acceptability | Interview | NR | NR |
| Young, 2014 [126] | Qualitative study | Rapid access to testing services | Clinic | USA | <200 | Feasibility | Other | NR | NR |
| d'Almeida, 2013 [127] | Hybrid study | Rapid access to testing services; NR | Hospital | France | >1000 | Acceptability; Fidelity | Quantitative analysis; Quantitative analysis | NR | NR |
| Baltzer Turje, 2013 [128] | Hybrid study | Individualized plan of care; Role of care navigators | Clinic; Community | Canada | <200 | Acceptability | Questionnaire | NR | NR |
| Bilardi, 2013 [129] | Qualitative study | Regular HIV testing for at risk groups; Rapid access to testing services | Clinic; Community | Australia | <200 | Acceptability | Interview | NR | NR |
| Burns, 2013 [130] | Qualitative study | Rapid access to testing services; Role of care navigators | Hospital | UK | <200 | Acceptability; Implementation costs | Questionnaire; Quantitative analysis | NR | NR |
| Ewing, 2013 [131] | Qualitative study | Rapid access to testing services; Role of care navigators | Clinic; GP | Australia | <200 | Feasibility | Quantitative analysis | NR | NR |
| Gaydos, 2013 [132] | Hybrid study | Rapid access to testing services | Hospital | USA | 200-500 | Acceptability; Feasibility | Questionnaire; Questionnaire | NR | NR |
| Hallum-Montes, 2013 [133] | Qualitative study | Role of care navigators | Hospital | USA | <200 | Sustainability | Interview; Framework | Grounded theory analysis by Strauss & Corbin | NR |
| Hennessey, 2013 [134] | Hybrid study | Rapid access to testing services; Role of care navigators | Hospital | USA | >1000 | Acceptability | Quantitative analysis | NR | Linkage to care |
| King, 2013 [135] | Hybrid study | Rapid access to testing services | Hospital; Clinic | UK | >1000 | Acceptability | Quantitative analysis | NR | Linkage to care |
| Knapp, 2013 [136] | Qualitative study | Rapid access to testing services | Clinic | USA | NR | Sustainability | Framework | ADAPTS - Assessment, Deliverables, Activate, Pretraining, Training, Sustainability | NR |
| Kurth, 2013 [137] | Hybrid study | Rapid access to testing services | Hospital | USA | 500-1000 | Acceptability; Implementation costs | Quantitative analysis; Quantitative analysis | NR | NR |
| Leber, 2013 [138] | Hybrid study | Rapid access to testing services | GP | UK | >1000 | Acceptability | Quantitative analysis | NR | Linkage to care |
| Lewis, 2013 [139] | Qualitative study | Rapid access to testing services | Clinic | Canada | 200-500 | Acceptability | Questionnaire | NR | Linkage to care |
| Read, 2013 [140] | Hybrid study | Rapid access to testing services; Regular HIV testing for at risk groups | Clinic | Australia | 200-500 | Acceptability; Sustainability | Questionnaire; Quantitative analysis | NR | NR |
| Seewald, 2013 [141] | Hybrid study | Regular HIV testing for at risk groups; Role of care navigators | Clinic | USA | >1000 | Acceptability | Quantitative analysis | NR | Linkage to care |
| Shahkolahi, 2013 [142] | Hybrid study | Rapid access to testing services | Hospital | USA | 200-500 | Acceptability | Quantitative analysis | NR | NR |
| Stenstrom, 2013 [143] | Hybrid study | Rapid access to testing services | Hospital | Canada | >1000 | Acceptability | Questionnaire | NR | NR |
| Williams, 2013 [144] | Hybrid study | Role of the pharmacist; Diagnosis & management of co-morbidities | Pharmacy; Hospital | USA | <200 | Acceptability | Other | NR | NR |
| Young, 2013 [145] | Hybrid study | Regular HIV testing for at risk groups; Role of care navigators | Community | USA | <200 | Acceptability; NR | Quantitative analysis; NR | NR | NR |

^†^N represents the total number of EBIs included in this review. n represents the number of publications in which these EBIs are evaluated. Please refer to the manuscript for a list of the references used.
