# Supplementary material for: A need for implementation science to optimise the use of evidence-based interventions in HIV care: A systematic literature review
Source: PLoS One. 2019 Aug 19;14(8):e0220060. doi: 10.1371/journal.pone.0220060 (PMC6699703; doi:10.1371/journal.pone.0220060)
Supplement: S1 File — (DOCX) [file pone.0220060.s004.docx]

**S1 File. List of Excluded Publications After Full-Text Screening [1-339].**

1. Ahmed S, Kim MH, Abrams EJ. Risks and benefits of lifelong antiretroviral treatment for pregnant and breastfeeding women: A review of the evidence for the Option B+ approach. Current Opinion in HIV and AIDS. 2013;8(5):474-89.
2. Alcaide ML, Feaster DJ, Duan R, Cohen S, DIaz C, Castro JG, et al. The incidence of Trichomonas vaginalis infection in women attenDing nine sexually transmitted diseases clinics in the USA. Sexually Transmitted Infections. 2016;92(1):58-62.
3. Aletraris L, Roman PM. Provision of onsite HIV Services in Substance Use Disorder Treatment Programs: A Longitudinal Analysis. Journal of substance abuse treatment. 2015;57:1-8.
4. Althoff AL, Zelenev A, Meyer JP, Fu J, Brown S-E, Vagenas P, et al. Correlates of retention in HIV care after release from jail: results from a multi-site study. AIDS and behavior. 2013;17 Suppl 2:S156-70.
5. Babalola S, Van Lith LM, Mallalieu EC, Packman ZR, Myers E, Ahanda KS, et al. A Framework for Health Communication Across the HIV Treatment Continuum. Journal of acquired immune deficiency syndromes (1999). 2017;74 Suppl 1:S5-S14.
6. Baggaley R, Dalal S, Johnson C, Macdonald V, Mameletzis l, Rodolph M, et al. Beyond the 90-90-90: refocusing HIV prevention as part of the global HIV response. Journal of the International AIDS Society. 2016;19(4):NA.
7. Bain LE, Nkoke C, Noubiap JJN. UNAIDS 90-90-90 targets to end the AIDS epidemic by 2020 are not realistic: comment on Can the UNAIDS 90-90-90 target be achieved? A systematic analysis of national HIV treatment cascades. BMJ global health. 2017;2(2):e000227.
8. Balán I, Frasca T, Ibitoye M, Dolezal C, Carballo-Diéguez A. Fingerprick Versus Oral Swab: Acceptability of Blood-Based Testing Increases If Other STIs Can Be Detected. AIDS and behavior. 2017;21(2):501-4.
9. Barbour W, Galbraith J, Rodgers J. Comparison of HIV risky behaviors and knowledge of adolescents and parents presenting to a pediatric E.D. Journal of Investigative Medicine. 2014;62(2):570.
10. Barnes M, Rohailla S, McDougall P, Baltzer Turje R, De Prinse K, Chan-Carusone S. Harm reduction from the perspective of health care providers: Benefits and challenges. Canadian Journal of Infectious Diseases and Medical Microbiology. 2015;26:85B.
11. Barr D, Odetoyinbo M, Mworeko L, Greenberg J. The leadership of communities in HIV service delivery. AIDS (London, England). 2015;29 Suppl 2:S121-7.
12. Bauman LJ, Braunstein S, Calderon Y, Chhabra R, Cutler B, Leider J, et al. Barriers and facilitators of linkage to HIV primary care in New York City. Journal of acquired immune deficiency syndromes (1999). 2013;64 Suppl 1:S20-6.
13. Becofsky K, Wing EJ, McCaffery J, Boudreau M, Wing RR. Randomized trial of behavioral weight loss for HIV-infected patients. Topics in Antiviral Medicine. 2017;25(1):297s-8s.
14. Bemelmans M, Baert S, Negussie E, Bygrave H, Biot M, Jamet C, et al. Sustaining the future of HIV counselling to reach 90-90-90: a regional country analysis. Journal of the International AIDS Society. 2016;19(2):NA.
15. Beres LK, Narasimhan M, Robinson J, Welbourn A, Kennedy CE. Non-specialist psychosocial support interventions for women living with HIV: A systematic review. AIDS Care - Psychological and Socio-Medical Aspects of AIDS/HIV. 2017;29(9):1079-87.
16. Bertelsen NS, Selden E, Krass P, Keatley ES, Keller A. Primary Care Screening Methods and Outcomes for Asylum Seekers in New York City. Journal of Immigrant and Minority Health. 2018;20(1):171-7.
17. Betancourt TS, Ng LC, Kirk CM, Brennan RT, Beardslee WR, Stulac S, et al. Family-based promotion of mental health in children affected by HIV: a pilot randomized controlled trial. Journal of Child Psychology and Psychiatry. 2017;58(8):922(9).
18. Bias TE, Venugopalan V, Berkowitz LB, Cha A. Incidence of antiretroviral drug interactions during hospital course: The role of a pharmacist-led antiretroviral stewardship program. Journal of Pharmacy Technology. 2014;30(2):48-53.
19. Biradavolu M, Jia Y, Withers K, Kapetanovic S. Factors Influencing the Delivery of HIV-Related Services to Severely Mentally Ill Individuals: The Provider's Perspective. Psychosomatics. 2016;57(1):64-70.
20. Bisson GP, Gupta A, Miyahara S, Sung X, Bao J, Fry CL, et al. Urine lam testing in advanced HIV-infected adults in a trial of empiric TB therapy. Topics in Antiviral Medicine. 2016;24(E-1):312-3.
21. Blais P, Sirivar S, Seto J. Supporting Implementation Research to Improve Coverage and Uptake of HIV Related Interventions. Journal of acquired immune deficiency syndromes (1999). 2017;75 Suppl 2:S109-S10.
22. Blank AE, Fletcher J, Verdecias N, Garcia I, Blackstock O, Cunningham C. Factors associated with retention and viral suppression among a cohort of HIV+ women of color. AIDS patient care and STDs. 2015;29 Suppl 1:S27-35.
23. Boehme AK, Davies SL, Moneyham L, Shrestha S, Schumacher J, Kempf M-C. A qualitative study on factors impacting HIV care adherence among postpartum HIV-infected women in the rural southeastern USA. AIDS care. 2014;26(5):574-81.
24. Booker CA, Flygare CT, Solomon L, Ball SW, Pustell MR, Bazerman LB, et al. Linkage to HIV care for jail detainees: findings from detention to the first 30 days after release. AIDS and behavior. 2013;17 Suppl 2:S128-36.
25. Bor J, Geldsetzer P, Venkataramani A, Bärnighausen T. Quasi-experiments to establish causal effects of HIV care and treatment and to improve the cascade of care. Current opinion in HIV and AIDS. 2015;10(6):495-501.
26. Borges AH, Neuhaus J, Babiker A, Wilkin TJ, Hoffmann C, Henry K, et al. Immediate art initiation reduces risk of infection-related cancer in HIV infection. Topics in Antiviral Medicine. 2016;24(E-1):64-5.
27. Borges ÁH, Neuhaus J, Babiker AG, Henry K, Jain MK, Palfreeman A, et al. Immediate Antiretroviral Therapy Reduces Risk of Infection-Related Cancer During Early HIV Infection. Clinical infectious diseases : an official publication of the Infectious Diseases Society of America. 2016;63(12):1668-76.
28. Bottero J, Boyd A, Gozlan J, Carrat F, Nau J, Pauti M-D, et al. Simultaneous human immunodeficiency virus-hepatitis B-hepatitis C point-of-care tests improve outcomes in linkage-to-care: Results of a randomized control trial in persons without healthcare coverage. Open Forum Infectious Diseases. 2015;2(4).
29. Bottero J BA, Gozlan J, Carrat F, Nau J, Pauti M-D, Rougier H, Girard P-M, Lacombe K. Simultaneous human immunodeficiency virus-hepatitis B-hepatitis C point-of-care tests improve outcomes in linkage-to-care: results of a randomized control trial in persons without healthcare coverage. Open forum infectious diseases. 2015;2(4) (no pagination).
30. Bouris A, Voisin D, Pilloton M, Flatt N, Eavou R, Hampton K, et al. Project nGage: Network Supported HIV Care Engagement for Younger Black Men Who Have Sex with Men and Transgender Persons. Journal of AIDS & clinical research. 2013;4.
31. Boyer CB, Walker BC, Chutuape KS, Roy J, Fortenberry JD. Creating systems change to support goals for HIV continuum of care: The role of community coalitions to reduce structural barriers for adolescents and young adults. J HIV AIDS Soc Services. 2016;15(2):158-79.
32. Brennan A, Browne JP, Horgan M. A systematic review of health service interventions to improve linkage with or retention in HIV care. AIDS Care - Psychological and Socio-Medical Aspects of AIDS/HIV. 2014;26(7):804-12.
33. Broaddus MR, Hanna CR, Schumann C, Meier A. she makes me feel that I'm not alone: Linkage to Care Specialists provide social support to people living with HIV. AIDS Care - Psychological and Socio-Medical Aspects of AIDS/HIV. 2015;27(9):1104-7.
34. Brown LK HW, Donenberg GR, DiClemente RJ, Lescano C, Lang DM, Crosby R, Barker D, Oster D. Project STYLE: a multisite RCT for HIV prevention among youths in mental health treatment. Psychiatric services (washington, DC). 2014;65(3):338-44.
35. Buchberg MK, Fletcher FE, Vidrine DJ, Levison J, Peters MY, Hardwicke R, et al. A mixed-methods approach to understanding barriers to postpartum retention in care among low-income, HIV-infected women. AIDS patient care and STDs. 2015;29(3):126-32.
36. Canidate S, Hart M. The Use of Avatar Counseling for HIV/AIDS Health Education: The Examination of Self-Identity in Avatar Preferences. Journal of medical Internet research. 2017;19(12):e365.
37. Capili B, Anastasi JK, Chang M, Ogedegbe O. Barriers and facilitators to engagement in lifestyle interventions among individuals with HIV. The Journal of the Association of Nurses in AIDS Care : JANAC. 2014;25(5):450-7.
38. Cargill VA. Linkage, engagement, and retention in HIV care among vulnerable populations: Im sick and tired of being sick and tired. Topics in antiviral medicine. 2013;21(4):133-7.
39. Carmona S, Peter T, Berrie L. HIV viral load scale-up: Multiple interventions to meet the HIV treatment cascade. Current Opinion in HIV and AIDS. 2017;12(2):157-64.
40. Chaiyachati KH, Ogbuoji O, Price M, Suthar AB, Negussie EK, Bärnighausen T. Interventions to improve adherence to antiretroviral therapy: A rapid systematic review. AIDS. 2014;28(SUPPL. 2):S187-S204.
41. Chibanda D. Depression and HIV: Integrated care towards 90-90-90. International Health. 2017;9(2):77-9.
42. Choi S, Boyle E, Kumar M, Cairney J, Krahn MD, Grootendorst P, et al. An examination of disparity in access to mental health services among people living with human immunodeficiency virus (HIV) and co-morbid depression in Ontario. Value in Health. 2014;17(3):A145.
43. Christopoulos KA RE, Tulsky J, Carrico AW, Moskowitz JT, Wilson L, Coffin LS, Falahati V, Akerley J, Hilton JF. A text messaging intervention to improve retention in care and virologic suppression in a U.S. urban safety-net HIV clinic: study protocol for the Connect4Care (C4C) randomized controlled trial. BMC infectious diseases. 2014;14:718.
44. Chuah FLH, Haldane VE, Cervero-Liceras F, Ong SE, Sigfrid LA, Murphy G, et al. Interventions and approaches to integrating HIV and mental health services: a systematic review. Health policy and planning. 2017;32(suppl_4):iv27-iv47.
45. Colasanti J, Goswami ND, Khoubian JJ, Pennisi E, Root C, Ziemer D, et al. The Perilous Road from HIV Diagnosis in the Hospital to Viral Suppression in the Outpatient Clinic. AIDS research and human retroviruses. 2016;32(8):729-36.
46. Connolly NB, Mellor J, Fothergill H, Schembri G, Babu C, McQuillan O. Retaining people living with HIV (PWH) in high-quality specialist care by means of a hub-and-spoke outreach clinic. HIV Medicine. 2013;14:20.
47. Corless IB, Wolf KA. Qualitative Caring and Quantitative Results: 90-90-90. The Journal of the Association of Nurses in AIDS Care : JANAC. 2016;27(6):752-4.
48. Cox J, Maurais E, Mejia P, Kelly-Shepard R, Fallu J, Brown M, et al. Feasibility of case management for HIV-infected patients with substance use disorders. Translating evaluation research into practice at the chronic viral illness service in montreal. Canadian Journal of Infectious Diseases and Medical Microbiology. 2015;26:76B.
49. Cox LE, Brennan-Ing M. Medical, Social and Supportive Services for Older Adults with HIV. Interdisciplinary topics in gerontology and geriatrics. 2017;42:204-21.
50. Crock EA, Miller C, McKenzie R, Burk N, Frecker J, Hall JE, et al. Emerging Needs of People Living With HIV Receiving Community-Based Nursing in an Australian Setting. The Journal of the Association of Nurses in AIDS Care : JANAC. 2017;28(4):644-58.
51. Darin KM, Klepser ME, Klepser DE, Klepser SA, Reeves A, Young M, et al. Pharmacist-provided rapid HIV testing in two community pharmacies. Journal of the American Pharmacists Association : JAPhA. 2015;55(1):81-8.
52. Darin KM, Scarsi KK, Klepser DG, Klepser SA, Reeves A, Young M, et al. Consumer interest in community pharmacy HIV screening services. Journal of the American Pharmacists Association : JAPhA. 2015;55(1):67-72.
53. Davies SH, Overholt SE, Hoffman ND. Ethics, emotional quotient, and interper-sonal connection: Peer identified characteristics for urban high school-based peer navigators. Journal of Adolescent Health. 2018;62(2):S127.
54. Davila JA, Miertschin N, Sansgiry S, Schwarzwald H, Henley C, Giordano TP. Centralization of HIV services in HIV-positive African-American and Hispanic youth improves retention in care. AIDS care. 2013;25(2):202-6.
55. De Rossi N, Dattner N, Cavassini M, Peters S, Hugli O, Darling KEA. Patient and doctor perspectives on HIV screening in the emergency department: A prospective cross-sectional study. PloS one. 2017;12(7):e0180389.
56. Del Rio C. HIV Infection in Hard-to-Reach Populations. Topics in antiviral medicine. 2016;24(2):86-9.
57. Delany-Moretlwe S, Cowan FM, Busza J, Bolton-Moore C, Kelley K, Fairlie L. Providing comprehensive health services for young key populations: needs, barriers and gaps. Journal of the International AIDS Society. 2015;18(2 Suppl 1):19833.
58. Dennis AC, Barrington C, Hino S, Gould M, Wohl D. 'You're in a world of chaos': Experiences accessing HIV care and adhering to medications after incarceration. J Assoc Nurses AIDS Care. 2015;26(5):542-5.
59. Dias S, Gama A, Pingarilho M, Simões D, Mendão L. Health Services Use and HIV Prevalence Among Migrant and National Female Sex Workers in Portugal: Are We Providing the Services Needed? AIDS and behavior. 2017;21(8):2316-21.
60. Ditekemena Dinanga J, Gill MM, Loando A, Nyombe C, Bakwalufu J, Mbonze N, et al. Early retention in antenatal care among HIV-positive women enrolled in the Option B+ programme in Kinshasa, DRC. Journal of the International AIDS Society. 2016;19:153.
61. Dombrowski JC, Simoni JM, Katz DA, Golden MR. Barriers to HIV Care and Treatment Among Participants in a Public Health HIV Care Relinkage Program. AIDS patient care and STDs. 2015;29(5):279-87.
62. Doyle JS, Bowring AL, Iser DM, Sasadeusz J, Roney J, O'Reilly M, et al. Suitability of HCV treatment in primary care settings for individuals with HIV/hepatitis C coinfection. Journal of Hepatology. 2017;66(1):S524-S5.
63. Draucker CB, Johnson DM, Johnson-Quay NL, Kadeba MT, Mazurczyk J, Zlotnick C. Rapid HIV testing and counseling for residents in domestic violence shelters. Women & Health. 2015;55(3):334(19).
64. Drew RS, Rice B, Rüütel K, Delpech V, Attawell KA, Hales DK, et al. HIV continuum of care in Europe and Central Asia. HIV Medicine. 2017;18(7):490-9.
65. Drummond KL, Painter JT, Curran GM, Stanley R, Gifford AL, Rodriguez-Barradas M, et al. HIV patient and provider feedback on a telehealth collaborative care for depression intervention. AIDS Care - Psychological and Socio-Medical Aspects of AIDS/HIV. 2017;29(3):290-8.
66. Duncombe C, Rosenblum S, Hellmann N, Holmes C, Wilkinson L, Biot M, et al. Reframing HIV care: putting people at the centre of antiretroviral delivery. Tropical medicine & international health : TM & IH. 2015;20(4):430-47.
67. Durbin A, Sirotich F, Lunsky Y, Roesslein K, Durbin J. Needs among persons with human immunodeficiency virus and intellectual and developmental disabilities in community mental health care: a cross-sectional study. Journal of Intellectual Disability Research. 2017;61(3):292(8).
68. Eaton EF, Saag MS, Mugavero M. Engagement in human immunodeficiency virus care: Linkage, retention, and antiretroviral therapy adherence. Infectious Disease Clinics of North America. 2014;28(3):355-69.
69. Eisenberg MM, Hennessy M, Coviello D, Hanrahan N, Blank MB. Coercion or Caring: The Fundamental Paradox for Adherence Interventions for HIV+ People with Mental Illness. AIDS and behavior. 2017;21(6):1530-9.
70. Engler K, Rollet K, Lessard D, Thomas R, Lebouché B. Ability of a rapid HIV testing site to attract and test vulnerable populations: a cross-sectional study on Actuel sur Rue. International journal of STD & AIDS. 2016;27(11):973-7.
71. Esposito-Smythers C, Hadley W, Curby TW, Brown LK. Randomized pilot trial of a cognitive-behavioral alcohol, self-harm, and HIV prevention program for teens in mental health treatment. Behaviour Research and Therapy. 2017;89:49(8).
72. Esposito-Smythers C HW, Curby TW, Brown LK. Randomized pilot trial of a cognitive-behavioral alcohol, self-harm, and HIV prevention program for teens in mental health treatment. Behaviour research and therapy. 2017;89:49-56.
73. Esteban-Vasallo MD, Morán-Arribas M, García-Riolobos C, Domínguez-Berjón MF, Rico-Bermejo J, Collado-González S, Jiménez-García R, Guionnet A, de la Fuente BP, El Kertat R, Coundoul A.Targeted rapid HIV testing in public primary care services in Madrid. Are we reaching the vulnerable populations? International Journal of Infectious Diseases. 2014;19:39(7).
74. Estem KS, Catania J, Klausner JD. HIV Self-Testing: a Review of Current Implementation and Fidelity. Current HIV/AIDS Reports. 2016;13(2):107-15.
75. F S. In early HIV infection, immediate vs deferred antiretroviral therapy reduced serious illnesses at 3 years. Annals of internal medicine. 2015;163(12):JC4-JC5.
76. Farley JE, Treston C. Nurses' Call to Action: Supporting a Strong Global HIV Nursing Workforce. The Journal of the Association of Nurses in AIDS Care : JANAC. 2016;27(6):741-4.
77. Farmer RE, Kounali D, Walker AS, Savovic J, Richards A, May MT, et al. Application of causal inference methods in the analyses of randomised controlled trials: A systematic review. Trials. 2018;19(1).
78. Fayorsey RN, Chege D, Wang C, Reidy W, Peters Z, Syengo M, et al. Mother infant retention for health (MIR4Health): Study design, adaptations, and challenges with PMTCT implementation science research. Journal of Acquired Immune Deficiency Syndromes. 2016;72:S137-S44.
79. Fernández-Balbuena S, de la Fuente L, Hoyos J, Rosales-Statkus ME, Barrio G, Belza M-J. Highly visible street-based HIV rapid testing: is it an attractive option for a previously untested population? A cross-sectional study. Sexually transmitted infections. 2014;90(2):112-8.
80. Fernàndez-López L, Reyes-Urueña J, Agustí C, Kustec T, Klavs I, Casabona C. The COBATEST network: A platform to perform monitoring and evaluation of HIV community-based testing practices in Europe and conduct operational research. AIDS Care - Psychological and Socio-Medical Aspects of AIDS/HIV. 2016;28:32-6.
81. Fernando S, McNeil R, Closson K, Samji H, Kirkland S, Strike C, et al. An integrated approach to care attracts people living with HIV who use illicit drugs in an urban centre with a concentrated HIV epidemic. Harm Reduction Journal. 2016;13(1).
82. Fonjungo PN, Boeras DI, Zeh C, Alexander H, Parekh BS, Nkengasong JN. Access and Quality of HIV-Related Point-of-Care Diagnostic Testing in Global Health Programs. Clinical infectious diseases : an official publication of the Infectious Diseases Society of America. 2016;62(3):369-74.
83. Fraisse T, Fourcade C, Brazes-Sanz J, Koumar Y, Lavigne JP, Sotto A, et al. A cross sectional survey of the barriers for implementing rapid HIV testing among French general practitioners. International journal of STD & AIDS. 2016;27(11):1005-12.
84. Freer J, Lascar M, Phiri E. Tailoring HIV testing in a setting of late HIV diagnosis: is the tide turning? British journal of hospital medicine (London, England : 2005). 2015;76(10):592-5.
85. Freese TE, Padwa H, Oeser BT, Rutkowski BA, Schulte MT. Real-World Strategies to Engage and Retain Racial-Ethnic Minority Young Men Who Have Sex with Men in HIV Prevention Services. AIDS patient care and STDs. 2017;31(6):275-81.
86. Frimpong JA, D'Aunno T, Helleringer S, Metsch LR. Low Rates of Adoption and Implementation of Rapid HIV Testing in Substance Use Disorder Treatment Programs. Journal of Substance Abuse Treatment. 2016;63:46-53.
87. Fritz CQ, Blevins M, Lindegren ML, Wools-Kaloutsian K, Musick BS, Cornell M, et al. Comprehensiveness of HIV care provided at global HIV treatment sites in the IeDEA consortium: 2009 and 2014. Journal of the International AIDS Society. 2017;20(1).
88. Gamble T, Branson B, Donnell D, Hall HI, King G, Cutler B, et al. Design of the HPTN 065 (TLC-Plus) study: A study to evaluate the feasibility of an enhanced test, link-to-care, plus treat approach for HIV prevention in the United States. Clinical Trials. 2017;14(4):322-32.
89. Ganguli I CJ, Reichmann WM, Losina E, Katz JN, Arbelaez C, Donnell-Fink LA, Walensky RP. Missed opportunities: refusal to confirm reactive rapid HIV tests in the emergency department. Plos one. 2013;8(1):e53408.
90. Garcia de Olalla P, Molas E, Barbera MJ, Martin S, Arellano E, Gosch M, et al. Effectiveness of a Pilot Partner Notification Program for New HIV Cases in Barcelona, Spain. PLoS ONE. 2015;10(4):NA.
91. Garcia J CP, Parker C, Hirsch JS. Passing the baton: community-based ethnography to design a randomized clinical trial on the effectiveness of oral pre-exposure prophylaxis for HIV prevention among Black men who have sex with men. Contemporary clinical trials. 2015;45(Pt B):244-51.
92. Garnett G. Delivery of HIV/STI prevention interventions: Prevention cascades. Sexually Transmitted Infections. 2015;91:A6.
93. Gelaude DJ, Hart J, Carey JW, Denson D, Erickson C, Klein C, et al. HIV Provider Experiences Engaging and Retaining Patients in HIV Care and Treatment: A Soft Place to Fall. The Journal of the Association of Nurses in AIDS Care : JANAC. 2017;28(4):491-503.
94. Geldsetzer P, Yapa HMN, Vaikath M, Ogbuoji O, Fox MP, Essajee SM, et al. A systematic review of interventions to improve postpartum retention of women in PMTCT and ART care. Journal of the International AIDS Society. 2016;19(1).
95. Genberg BL, Shangani S, Sabatino K, Rachlis B, Wachira J, Braitstein P, et al. Improving Engagement in the HIV Care Cascade: A Systematic Review of Interventions Involving People Living with HIV/AIDS as Peers. AIDS and behavior. 2016;20(10):2452-63.
96. Gennimata D, Malliarou M, Marini F, Chini M, Pitsounis N, Lazanas M. Improvement of Health Care Services for ambulatory HIV patients: Preliminary data. European Journal of Hospital Pharmacy. 2014;21:A195-A6.
97. Geretti AM, Loutfy M, D'Arminio Monforte A, Latysheva I, Pérez Elías MJ, Rymer J, et al. Out of focus: tailoring the cascade of care to the needs of women living with HIV. HIV medicine. 2017;18 Suppl 2:3-17.
98. Gillet C, Darling KEA, Senn N, Cavassini M, Hugli O. Targeted versus non-targeted HIV testing offered via electronic questionnaire in a Swiss emergency department: A randomized controlled study. PLoS ONE. 2018;13(3).
99. Gillis JR, Umana M, Tharao W, Muchenje M, Chikermane V, Mudiwa D, et al. HIV health literacy for racialized HIV-positive women: What we know and what we need to know. Canadian Journal of Infectious Diseases and Medical Microbiology. 2014;25:13A.
100. Goldman TR. Living with HIV and growing old. Health affairs (Project Hope). 2014;33(3):359-61.
101. Golin CE, Knight K, Carda-Auten J, Gould M, Groves J, L White B, et al. Individuals motivated to participate in adherence, care and treatment (imPACT): development of a multi-component intervention to help HIV-infected recently incarcerated individuals link and adhere to HIV care. BMC public health. 2016;16:935.
102. Golin CE KK, Carda-Auten J, Gould M, Groves J, L White B, Bradley-Bull S, Amola K, Fray N, Rosen DL, Mugavaro MJ, Pence BW, Flynn PM, Wohl D. Individuals motivated to participate in adherence, care and treatment (imPACT): development of a multi-component intervention to help HIV-infected recently incarcerated individuals link and adhere to HIV care. BMC public health. 2016;16:935.
103. Gordon MS, Kinlock TW, McKenzie M, Wilson ME, Rich JD. Rapid HIV testing for individuals on probation/parole: outcomes of an intervention trial. AIDS and behavior. 2013;17(6):2022-30.
104. Gordon MS KT, McKenzie M, Wilson ME, Rich JD. Rapid HIV testing for individuals on probation/parole: outcomes of an intervention trial. AIDS and behavior. 2013;17(6):2022-30.
105. Granich R, Williams B, Montaner J, Zuniga JM. 90-90-90 and ending AIDS: necessary and feasible. Lancet (London, England). 2017;390(10092):341-3.
106. Greene E, Pack A, Stanton J, Shelus V, Tolley EE, Taylor J, et al. It Makes You Feel Like Someone Cares acceptability of a financial incentive intervention for HIV viral suppression in the HPTN 065 (TLC-Plus) study. PLoS ONE. 2017;12(2):e0170686.
107. Gregoriano C, Henny-Reinalter S, Maier S, Flamm AL, Dieterle T, Arnet I, Hersberger KE, Leuppi JD. ESCP International Workshop Medication Adherence from Theory to Daily Patient Care. International Journal of Clinical Pharmacy. 2016;38(5).
108. Grelotti DJ, Hammer GP, Dilley JW, Karasic DH, Sorensen JL, Bangsberg DR, et al. Does substance use compromise depression treatment in persons with HIV? Findings from a randomized controlled trial †. AIDS Care - Psychological and Socio-Medical Aspects of AIDS/HIV. 2017;29(3):273-9.
109. Guaraldi G, Palella FJ, Jr. Clinical implications of aging with HIV infection: perspectives and the future medical care agenda. AIDS (London, England). 2017;31 Suppl 2:S129-S35.
110. Guerrero EG, Fenwick K, Kong Y, Grella C, D'Aunno T. Paths to improving engagement among racial and ethnic minorities in addiction health services. Substance abuse treatment, prevention, and policy. 2015;10:40.
111. Guise A. Next generation of comprehensive HIV prevention. The Lancet. 2016;387(10036):2377-8.
112. Gupta S, Granich R. National HIV Care Continua for Key Populations. Journal of the International Association of Providers of AIDS Care. 2017;16(2):125-32.
113. Habel MA, Becasen JS, Dittus PJ. The state of sexual health services at U.S. colleges & universities. Journal of Adolescent Health. 2015;56(2):S74-S5.
114. Hahm HC, Chang ST-H, Lee GY, Tagerman MD, Lee CS, Trentadue MP, et al. Asian Women's Action for Resilience and Empowerment Intervention: Stage I Pilot Study. Journal of cross-cultural psychology. 2017;48(10):1537-53.
115. Haldane V, Cervero-Liceras F, Chuah FL, Ong SE, Murphy G, Sigfrid L, et al. Integrating HIV and substance use services: a systematic review. Journal of the International AIDS Society. 2017;20(1):21585.
116. Haldane V, Legido-Quigley H, Chuah FLH, Sigfrid L, Murphy G, Ong SE, et al. Integrating cardiovascular diseases, hypertension, and diabetes with HIV services: a systematic review. AIDS Care - Psychological and Socio-Medical Aspects of AIDS/HIV. 2018;30(1):103-15.
117. Herbert G, Sutton E, Burden S, Lewis S, Thomas S, Ness A, et al. Healthcare professionals views of the enhanced recovery after surgery programme: a qualitative investigation. BMC Health Services Research. 2017;17.
118. Hickey MD, Odeny TA, Petersen M, Neilands TB, Padian N, Ford N, et al. Specification of implementation interventions to address the cascade of HIV care and treatment in resource-limited settings: a systematic review. Implementation science : IS. 2017;12(1):102.
119. Higa DH, Crepaz N, Mullins MM. Identifying Best Practices for Increasing Linkage to, Retention, and Re-engagement in HIV Medical Care: Findings from a Systematic Review, 1996-2014. AIDS and behavior. 2016;20(5):951-66.
120. Hile SJ, Feldman MB, Raker AR, Irvine MK. Identifying Key Elements to Inform the Development of an HIV Health Behavior Maintenance Intervention. American journal of health promotion : AJHP. 2018;32(1):48-58.
121. Hill M, Huff A, Chumbler N. Variation in Networks and Forms of Support for Care-Seeking Across the HIV Care Continuum in the Rural Southeastern United States. The Journal of rural health : official journal of the American Rural Health Association and the National Rural Health Care Association. 2018;34(1):71-9.
122. Holt M, Lea T, Murphy DA, Ellard J, Rosengarten M, Kippax SC. Australian Gay and Bisexual Men's Attitudes to HIV Treatment as Prevention in Repeated, National Surveys 2011-2013. PLoS ONE. 2014;9(11):NA.
123. Holtzman CW, Brady KA, Yehia BR. Retention in care and medication adherence: current challenges to antiretroviral therapy success. Drugs. 2015;75(5):445-54.
124. Hoos D, El-Sadr WM, Dehne K-L. Getting the balance right: Scaling-up treatment and prevention. Global Public Health. 2017;12(4):483-97.
125. Hoy JF, Grund B, Roediger M, Schwartz AV, Shepherd J, Avihingsanon A, et al. Immediate Initiation of Antiretroviral Therapy for HIV Infection Accelerates Bone Loss Relative to Deferring Therapy: Findings from the START Bone Mineral Density Substudy, a Randomized Trial. Journal of Bone and Mineral Research. 2017;32(9):1945-55.
126. Hoyos J, Fernández-Balbuena S, de la Fuente L, Sordo L, Ruiz M, Barrio G, et al. Never tested for HIV in Latin-American migrants and Spaniards: prevalence and perceived barriers. Journal of the International AIDS Society. 2013;16:18560.
127. Hoyos Miller J, Fernández-Balbuena S, Belza Egozcue MJ, García De Olalla P, Pulido Manzanero J, Molist Señe G, et al. Time devoted to pre- and post-HIV test counselling in different health services according to participants of a rapid testing program in Madrid, Spain. Enfermedades Infecciosas y Microbiologia Clinica. 2014;32(2):82-6.
128. Hughes A, Scheer S. The causal effect of depression on viral suppression among adults in HIV care. Topics in Antiviral Medicine. 2017;25(1):391s.
129. Hurt CB, Soni K, Miller WC, Hightow-Weidman LB. Human Immunodeficiency Virus Testing Practices and Interest in Self-Testing Options Among Young, Black Men Who Have Sex With Men in North Carolina. Sexually transmitted diseases. 2016;43(9):587-93.
130. Immune System Diseases and Conditions; Investigators at Massachusetts General Hospital Have Reported New Data on HIV/AIDS. Health Insurance Week. 2014.
131. Iwuji C, Newell M-L. Towards control of the global HIV epidemic: Addressing the middle-90 challenge in the UNAIDS 90-90-90 target. PLoS medicine. 2017;14(5):e1002293.
132. Jamil MS, Prestage G, Fairley CK, Smith KS, Kaldor JM, Grulich AE, et al. Rationale and design of FORTH: a randomised controlled trial assessing the effectiveness of HIV self-testing in increasing HIV testing frequency among gay and bisexual men. BMC infectious diseases. 2015;15:561.
133. Jean-Philippe P, Spiegel H, Gnanashanmugam D, Fitzgibbon J, D'Souza P, Crawford KW, et al. HIV birth testing and linkage to care for HIV-infected infants. AIDS. 2017;31(13):1797-807.
134. Jeffries C, Ross P, Matoff-Stepp S, Thompson R, Harris JL, Uhrig JD, et al. Ucare4life: Mobile texting to improve HIV care continuum outcomes for minority youth. Topics in Antiviral Medicine. 2016;24(E-1):427.
135. Johnson CC, Dalal S, Baggaley R, Taegtmeyer M, Fonner V, Sands A, et al. A public health approach to addressing and preventing misdiagnosis in the scale-up of HIV rapid testing programmes:. Journal of the International AIDS Society. 2017;20.
136. Johnson CC, Fonner V, Sands A, Ford N, Obermeyer CM, Tsui S, et al. To err is human, to correct is public health: A systematic review examining poor quality testing and misdiagnosis of HIV status. Journal of the International AIDS Society. 2017;20.
137. Jones D, Chakhtoura N, Cook R. Reproductive and maternal healthcare needs of HIV infected women. Current HIV/AIDS reports. 2013;10(4):333-41.
138. Jones J, Taylor BS, Tieu H-V, Wilkin TJ. CROI 2017: Advances in antiretroviral therapy. Topics in Antiviral Medicine. 2017;25(2):51-67.
139. Jongbloed K, Parmar S, van der Kop M, Spittal PM, Lester RT. Recent Evidence for Emerging Digital Technologies to Support Global HIV Engagement in Care. Current HIV/AIDS reports. 2015;12(4):451-61.
140. Joseph Davey D, Myer L, Bukusi E, Ramogola-Masire D, Kilembe W, Klausner JD. Integrating Human Immunodeficiency Virus and Reproductive, Maternal and Child, and Tuberculosis Health Services Within National Health Systems. Current HIV/AIDS reports. 2016;13(3):170-6.
141. Judd A, Sohn AH, Collins IJ. Interventions to improve treatment, retention and survival outcomes for adolescents with perinatal HIV-1 transitioning to adult care: moving on up. Current opinion in HIV and AIDS. 2016;11(5):477-86.
142. Ka'opua LSI, Diaz TP, Park SH, Bowen T, Patrick K, Tamang S, et al. Colorectal cancer screening at the nexus of HIV minority statuses, and cultural safety. American Journal of Health Education. 2014;45(1):42(10).
143. Kapalko A, Lalley-Chareczko L, Fibbi MF, Mounzer K. Evaluation of an integrated HIV/HCV care model in an Urban, low-income, HIV/HCV co-infection clinic. Hepatology. 2015;62:770A.
144. Kaplan JE, Vallabhaneni S, Smith RM, Chideya-Chihota S, Chehab J, Park B. Cryptococcal antigen screening and early antifungal treatment to prevent cryptococcal meningitis: a review of the literature. Journal of acquired immune deficiency syndromes (1999). 2015;68 Suppl 3:S331-9.
145. Katz D GM, Hughes J, Farquhar C, Stekler J. HIV self-testing increases HIV testing frequency among highrisk men who have sex with men: a randomized controlled trial. Journal of the international AIDS society. 2015;18:95.
146. Kaye DL. Improving primary care access and quality of care for children with mental health needs: The child and adolescent psychiatry for primary care cap PC experience. Journal of the American Academy of Child and Adolescent Psychiatry. 2017;56(10):S132.
147. Kazanjian P. UNAIDS 90-90-90 Campaign to End the AIDS Epidemic in Historic Perspective. Milbank Quarterly. 2017;95(2):408-39.
148. Kempf M-C, Huang C-H, Savage R, Safren SA. Technology-Delivered Mental Health Interventions for People Living with HIV/AIDS (PLWHA): a Review of Recent Advances. Current HIV/AIDS Reports. 2015;12(4):472(9).
149. Kennedy CE, Haberlen SA, Narasimhan M. Integration of sexually transmitted infection (STI) services into HIV care and treatment services for women living with HIV: A systematic review. BMJ Open. 2017;7(6).
150. Kennedy CE, Yeh PT, Johnson C, Baggaley R. Should trained lay providers perform HIV testing? A systematic review to inform World Health Organization guidelines. AIDS Care - Psychological and Socio-Medical Aspects of AIDS/HIV. 2017;29(12):1473-9.
151. Kenya S, Okoro I, Wallace K, Carrasquillo O, Prado G. Strategies to Improve HIV Testing in African Americans. The Journal of the Association of Nurses in AIDS Care : JANAC. 2015;26(4):357-67.
152. Kessler J, Myers JE, Nucifora KA, Mensah N, Kowalski A, Sweeney M, et al. Averting HIV Infections in New York City: A Modeling Approach Estimating the Future Impact of Additional Behavioral and Biomedical HIV Prevention Strategies. PLoS ONE. 2013;8(9):e73269.
153. Kibicho J, Dilworth T, Owczarzak J. Community-based pharmacists' perceptions of physician collaboration and adherence promotion activities targeting persons living with HIV. Value in Health. 2016;19(3):A221.
154. Kidsley S, Williams S, Scholfield C. Improving access to better sexual health through the introduction of an on-line sexual transmitted infection home sampling kit service. BJOG: An International Journal of Obstetrics and Gynaecology. 2017;124:93.
155. Kielly J, Kelly DV, Asghari S, Burt K, Biggin J. Patient satisfaction with chronic HIV care provided through an innovative pharmacist/nurse-managed clinic and a multidisciplinary clinic. Canadian Pharmacists Journal. 2017;150(6):397-406.
156. Kisesa A, Chamla D. Getting to 90-90-90 targets for children and adolescents HIV in low and concentrated epidemics: Bottlenecks, opportunities, and solutions. Current Opinion in HIV and AIDS. 2016;11(1):S1-S5.
157. Ko N-Y, Liu H-Y, Lai Y-Y, Pai Y-H, Ko W-C. Case management interventions for HIV-infected individuals. Current HIV/AIDS reports. 2013;10(4):390-7.
158. Koester KA, Collins SP, Fuller SM, Galindo GR, Gibson S, Steward WT. Sexual healthcare preferences among gay and bisexual men: a qualitative study in San Francisco, California. PloS one. 2013;8(8):e71546.
159. Koester KA, Fuller SM, Maiorana A, Steward WT, Zamudio-Haas S, Xavier J, et al. Implementing Multi-Level Interventions to Improve HIV Testing, Linkage-to-and Retention-in-Care Interventions. Journal of health care for the poor and underserved. 2016;27(3):1234-51.
160. Kripke K, Reed J, Hankins C, Smiley G, Laube C, Njeuhmeli E. Correction: Impact and Cost of Scaling Up Voluntary Medical Male Circumcision for HIV Prevention in the Context of the New 90-90-90 HIV Treatment Targets. PloS one. 2016;11(12):e0169500.
161. Kudryashova Hernandez L. One-stop shop service delivery model: Integrating prevention interventions with HIV Care/Treatment services in a community-based medical home setting. Sexually Transmitted Infections. 2013;89.
162. Kufa T, Hippner P, Charalambous S, Kielmann K, Vassall A, Churchyard GJ, et al. A cluster randomised trial to evaluate the effect of optimising TB/HIV integration on patient level outcomes: The merge trial protocol. Contemporary Clinical Trials. 2014;39(2):280-7.
163. Kunisaki KM, Niewoehner DE, Collins G, Aagaard B, Atako NB, Bakowska E, et al. Pulmonary effects of immediate versus deferred antiretroviral therapy in HIV-positive individuals: a nested substudy within the multicentre, international, randomised, controlled Strategic Timing of Antiretroviral Treatment (START) trial. The Lancet Respiratory medicine. 2016;4(12):980-9.
164. Kunisaki KM ND, Collins G, Aagaard B, Atako NB, Bakowska E, Clarke A, Corbelli GM, Ekong E, Emery S, Finley EB, Florence E, Infante RM, Kityo CM, Madero JS, Nixon DE, Tedaldi E, Vestbo J, Wood R, Connett JE. Pulmonary effects of immediate versus deferred antiretroviral therapy in HIV-positive individuals: a nested substudy within the multicentre, international, randomised, controlled Strategic Timing of Antiretroviral Treatment (START) trial. The lancet respiratory medicine. 2016;4(12):980-9.
165. Kuo I, Phillips GL, Magnus M, Opoku J, Rawls AL, Peterson J, et al. Willingness to use pre-exposure prophylaxis among community-recruited injection drug users. Topics in Antiviral Medicine. 2014;22:501.
166. Kurth A, Kuo I, Peterson J, Azikiwe N, Bazerman L, Cates A, et al. Information and Communication Technology to Link Criminal Justice Reentrants to HIV Care in the Community. AIDS research and treatment. 2013;2013:547381.
167. Kuttner-May S, Kroenke S, Muenstermann D, Lucht A. HIV-and syphilis-counselling and-testing in the public health service in North Rhine-Westphalia (NRW). International Journal of Medical Microbiology. 2015;305:8-9.
168. Leber W MH, Anderson J, Marlin N, Santos AC, Bremner S, Boomla K, Kerry S, Millett D, Mguni S, Creighton S, Figueroa J, Ashcroft R, Hart G, Delpech V, Brown A, Rooney G, Sampson M, Martineau A, Terris-Prestholt F, Griffiths C. Promotion of rapid testing for HIV in primary care (RHIVA2): a cluster-randomised controlled trial. The lancet HIV. 2015;2(6):e229-35.
169. Leblanc J, Rousseau A, Hejblum G, Durand-Zaleski I, de Truchis P, Lert F, et al. The impact of nurse-driven targeted HIV screening in 8 emergency departments: Study protocol for the DICI-VIH cluster-randomized two-period crossover trial. BMC Infectious Diseases. 2016;16(1).
170. Leblanc LJ JC, Fossoux N, Lancien C, Bastide T, Verbrugghe R, Jauneau C, Piquet H, Cremieux A-C, Simon T. Effectiveness of nurse-driven HIV screening targeting key populations in emergency departments in metropolitan Paris: the anrs dici-vih cluster-randomized two-period crossover tria. Clinical therapeutics Conference: 13th congress of the european association for clinical pharmacology and therapeutics, EACPT 2017 Czech republic. 2017;39(8 Supplement 1):e5.
171. LeCroix RH, Goodrum NM, Hufstetler S, Armistead LP. Community Data Collection with Children of Mothers Living with HIV: Boundaries of the Researcher Role. American journal of community psychology. 2017;60(3-4):368-74.
172. Lee L, Yehia BR, Gaur AH, Rutstein R, Gebo K, Keruly JC, et al. The Impact of Youth-Friendly Structures of Care on Retention Among HIV-Infected Youth. AIDS patient care and STDs. 2016;30(4):170-7.
173. Lester RT. Using technology to improve adherence. Topics in Antiviral Medicine. 2017;25(1):40s-1s.
174. Leukefeld CG, Cawood M, Wiley T, Robertson AA, Fisher JH, Arrigona N, et al. The Benefits of Community and Juvenile Justice Involvement in Organizational Research. Journal of juvenile justice. 2017;6(1):112-24.
175. Li AT-W, Wales J, Wong JP-H, Owino M, Perreault Y, Miao A, et al. Changing access to mental health care and social support when people living with HIV/AIDS become service providers. AIDS Care - Psychological and Socio-Medical Aspects of AIDS/HIV. 2015;27(2):176-81.
176. Lifson AR, Grund B, Gardner EM, Kaplan R, Denning E, Engen N, et al. Improved quality of life with immediate versus deferred initiation of antiretroviral therapy in early asymptomatic HIV infection. AIDS. 2017;31(7):953-63.
177. Lodi S, Sharma S, Lundgren JD, Phillips AN, Cole SR, Logan R, et al. The per-protocol effect of immediate versus deferred antiretroviral therapy initiation. AIDS. 2016;30(17):2659-63.
178. Lorente N, Preau M, Vernay-Vaisse C, Mora M, Blanche J, Otis J, et al. Expanding Access to Non-Medicalized Community-Based Rapid Testing to Men Who Have Sex with Men: An Urgent HIV Prevention Intervention (The ANRS-DRAG Study). PLoS ONE. 2013;8(4).
179. Lovejoy TI, Heckman TG. Telephone-administered motivational interviewing and behavioral skills training to reduce risky sexual behavior in HIV-positive late middle-age and older adults. Cognitive and Behavioral Practice. 2014;21(2):224-36.
180. Lovejoy TI HT, Sikkema KJ, Hansen NB, Kochman A. Changes in sexual behavior of HIV-infected older adults enrolled in a clinical trial of standalone group psychotherapies targeting depression. AIDS and behavior. 2015;19(1):1-8.
181. Lundgren JD, Babiker AG, Gordin F, Emery S, Grund B, Sharma S, et al. Initiation of antiretroviral therapy in early asymptomatic HIV infection. New England Journal of Medicine. 2015;373(9):795-807.
182. Luo C, Hirnschall G, Rodrigues J, Romano S, Essajee S, Rogers B, et al. Translating Technical Support Into Country Action: The Role of the Interagency Task Team on the Prevention and Treatment of HIV Infection in Pregnant Women, Mothers, and Children in the Global Plan Era. Journal of acquired immune deficiency syndromes (1999). 2017;75 Suppl 1:S7-S16.
183. MacPherson P, Munthali C, Ferguson J, Armstrong A, Kranzer K, Ferrand RA, et al. Service delivery interventions to improve adolescents' linkage, retention and adherence to antiretroviral therapy and HIV care. Tropical medicine & international health : TM & IH. 2015;20(8):1015-32.
184. Manteuffel J, Markowitz N, Ham DC, Slezak M, Perrotta G, Peters PJ, et al. Implementation of an emergency department syphilis and HIV point-of-care screening process during an outbreak of syphilis in Detroit, MI in collaboration with the infectious disease department and the centers for disease control and prevention. Annals of Emergency Medicine. 2016;68(4):S148.
185. Marcus JL, Volk JE, Pinder J, Liu AY, Bacon O, Hare CB, et al. Successful Implementation of HIV Preexposure Prophylaxis: Lessons Learned From Three Clinical Settings. Current HIV/AIDS Reports. 2016;13(2):116-24.
186. Markwick N, Ti L, Callon C, Feng C, Wood E, Kerr T. Willingness to engage in peer-delivered HIV voluntary counselling and testing among people who inject drugs in a Canadian setting. Journal of epidemiology and community health. 2014;68(7):675-8.
187. Marshall N, Harvey S, Naous N, Khonyongwa K, Okoli C, Odejide O, et al. Pharmacist interventions on home delivery prescriptions in three London HIV outpatient clinics. HIV Medicine. 2013;14:16.
188. Marukutira T, Stoové M, Lockman S, Mills LA, Gaolathe T, Lebelonyane R, et al. A tale of two countries: progress towards UNAIDS 90-90-90 targets in Botswana and Australia. Journal of the International AIDS Society. 2018;21(3).
189. Marzan-Rodriguez M, Varas-Diaz N, Neilands T. Qualitative contributions to a randomized controlled trial addressing HIV/AIDS-stigma in medical students. The Qualitative Report. 2015;20(12):2013(13).
190. Mateo-Urdiales A, Johnson S, Nachega JB, Eshun-Wilson I. Rapid initiation of antiretroviral therapy for people living with HIV. Cochrane Database of Systematic Reviews. 2018;2018(2).
191. Maulsby C, Sacamano P, Jain KM, Enobun B, Brantley ML, Kim H-Y, et al. Barriers and Facilitators to the Implementation of a National HIV Linkage, Re-Engagement, and Retention in Care Program. AIDS education and prevention : official publication of the International Society for AIDS Education. 2017;29(5):443-56.
192. Mayo NE BM-J, Fellows LK. Understanding and optimizing brain health in HIV now: protocol for a longitudinal cohort study with multiple randomized controlled trials. BMC neurology. 2016;16(1).
193. McAdams R, Carroll L, Clutterbuck D, Coia N, Milne D, Morrison C, et al. National health service greater glasgow and clyde and national health service lothian HIV prevention needs assessment: Risk factors in men who have sex with men diagnosed with rectal gonorrhoea or chlamydia. International Journal of STD and AIDS. 2013;24:30.
194. McBrien KA, Ivers N, Barnieh L, Bailey JJ, Lorenzetti DL, Nicholas D, et al. Patient navigators for people with chronic disease: A systematic review. PLoS ONE. 2018;13(2):e0191980.
195. McCullagh C, Quinn K, Voisin DR, Schneider J. A longitudinal examination of factors associated with social support satisfaction among HIV-positive young Black men who have sex with men. AIDS Care - Psychological and Socio-Medical Aspects of AIDS/HIV. 2017;29(12):1598-604.
196. McMahon JM, Pouget ER, Tortu S, Volpe EM, Torres L, Rodriguez W. Couple-based HIV counseling and testing: a risk reduction intervention for US drug-involved women and their primary male partners. Prevention science : the official journal of the Society for Prevention Research. 2015;16(2):341-51.
197. McMahon JM PE, Tortu S, Volpe EM, Torres L, Rodriguez W. Couple-based HIV counseling and testing: a risk reduction intervention for US drug-involved women and their primary male partners. Prevention science : the official journal of the society for prevention research 16 (2) (pp 341-351), 2015 Date of publication: 01 feb 2015. 2015;16(2):341-51.
198. McMullen H, Griffiths C, Leber W, Greenhalgh T. Explaining high and low performers in complex intervention trials: A new model based on diffusion of innovations theory. Trials. 2015;16(1).
199. McNicholl IR, Gandhi M, Hare CB, Greene M, Pierluissi E. A Pharmacist-Led Program to Evaluate and Reduce Polypharmacy and Potentially Inappropriate Prescribing in Older HIV-Positive Patients. Pharmacotherapy. 2017;37(12):1498-506.
200. McNulty MC, Schneider JA. Care continuum entry interventions: Seek and test strategies to engage persons most impacted by HIV within the United States. AIDS. 2018;32(4):407-17.
201. McVeigh J, Hearne E, Bates G, Van Hout MC. Community pharmacist experiences of providing needle and syringe programmes in Ireland. Research in social & administrative pharmacy : RSAP. 2017;13(4):767-77.
202. Medline A, Daniels J, Marlin R, Young S, Wilson G, Huang E, et al. HIV Testing Preferences Among MSM Members of an LGBT Community Organization in Los Angeles. The Journal of the Association of Nurses in AIDS Care : JANAC. 2017;28(3):363-71.
203. Meyers K, Golub SA. Planning ahead for implementation of long-acting HIV prevention: Challenges and opportunities. Current Opinion in HIV and AIDS. 2015;10(4):290-5.
204. Meyerson BE, Ryder PT, Von Hippel C, Coy K. More than just selling the test: Pharmacist opinion about the sale of over the counter HIV rapid tests. Sexually Transmitted Infections. 2013;89.
205. Miners AH, Llewellyn CD, Cooper VL, Youssef E, Pollard AJ, Lagarde M, et al. A discrete choice experiment to assess people living with HIV's (PLWHIV's) preferences for GP or HIV clinic appointments. Sexually transmitted infections. 2017;93(2):105-11.
206. Mitchell JW, Sullivan PS. Brief Report: Relationship and Demographic Factors Associated With Willingness to Use an In-Home Rapid HIV Test to Screen Potential Sex Partners Among a US Sample of HIV-Negative and HIV-Discordant Male Couples. Journal of acquired immune deficiency syndromes (1999). 2015;69(2):252-6.
207. Mitchell JW, Sullivan PS. HIV-negative partnered men's attitudes toward using an in-home rapid HIV test and associated factors among a sample of US HIV-Negative and HIV-discordant male couples. Sexually transmitted diseases. 2015;42(3):123-8.
208. Molina J-M, Grund B, Gordin F, Williams I, Schechter M, Losso M, et al. Which HIV-infected adults with high CD4 T-cell counts benefit most from immediate initiation of antiretroviral therapy? A post-hoc subgroup analysis of the START trial. The lancet HIV. 2018.
209. Moore RC, Marquine MJ, Straus E, Depp CA, Moore DJ, Schiehser DM, et al. Predictors and Barriers to Mental Health Treatment Utilization Among Older Veterans Living With HIV. The primary care companion for CNS disorders. 2017;19(1).
210. Moosavy SH, Davoodian P, Nazarnezhad MA, Nejatizaheh A, Eftekhar E, Mahboobi H. Epidemiology, transmission, diagnosis, and outcome of Hepatitis C virus infection. Electronic physician. 2017;9(10):5646-56.
211. Morales-Aleman MM, Sutton MY. Hispanics/Latinos and the HIV continuum of care in the Southern USA: a qualitative review of the literature, 2002-2013. AIDS care. 2014;26(12):1592-604.
212. Moscou-Jackson G, Commodore-Mensah Y, Farley J, DiGiacomo M. Smoking-Cessation Interventions in People Living With HIV Infection: A Systematic Review. Journal of the Association of Nurses in AIDS Care. 2014;25(1):32-45.
213. Mugwanya KK, Donnell D, Celum C, Thomas KK, Ndase P, Mugo N, et al. Sexual behaviour of heterosexual men and women receiving antiretroviral pre-exposure prophylaxis for HIV prevention: a longitudinal analysis. The Lancet Infectious Diseases. 2013;13(12):1021-8.
214. Mukumbang FC, Van Belle S, Marchal B, van Wyk B. Exploring 'generative mechanisms' of the antiretroviral adherence club intervention using the realist approach: a scoping review of research-based antiretroviral treatment adherence theories. BMC public health. 2017;17(1):385.
215. Murray A, Toledo L, Brown EE, Sutton MY. We as Black Men Have to Encourage Each other: Facilitators and Barriers Associated with HIV Testing among Black/African American Men in Rural Florida. Journal of health care for the poor and underserved. 2017;28(1):487-98.
216. Murray K, Cummins D, Bloom K. Developing a protocol for people living with HIV entering residential aged care facilities. Australian nursing & midwifery journal. 2014;21(11):34-6.
217. Muyoyeta M, Moyo M, Kasese N, Ndhlovu M, Milimo D, Mwanza W, et al. Implementation research to inform the use of xpert MTB/RIF in primary health care facilities in high TB and HIV settings in resource constrained settings. PLoS ONE. 2015;10(6).
218. Myers JE, Bodach S, Cutler BH, Shepard CW, Philippou C, Branson BM. Acceptability of home self-tests for HIV in New York City, 2006. American journal of public health. 2014;104(12):e46-8.
219. Myers JE, El-Sadr Davis OY, Weinstein ER, Remch M, Edelstein A, Khawja A, et al. Availability, Accessibility, and Price of Rapid HIV Self-Tests, New York City Pharmacies, Summer 2013. AIDS and behavior. 2017;21(2):515-24.
220. Natoli L, Guy RJ, Shephard M, Causer L, Badman SG, Hengel B, et al. I Do Feel Like a Scientist at Times: A Qualitative Study of the Acceptability of Molecular Point-Of-Care Testing for Chlamydia and Gonorrhoea to Primary Care Professionals in a Remote High STI Burden Setting. PLoS ONE. 2015;10(12):e0145993.
221. Navarra A-MD, Gwadz MV, Whittemore R, Bakken SR, Cleland CM, Burleson W, et al. Health Technology-Enabled Interventions for Adherence Support and Retention in Care Among US HIV-Infected Adolescents and Young Adults: An Integrative Review. AIDS and behavior. 2017;21(11):3154-71.
222. Nunn A, Chan P, Towey C, Poceta J, Feller S, Trooksin S. Acceptability & affordability of self HIV testing in an urban neighborhood with 3% seroprevalencee. Topics in Antiviral Medicine. 2014;22:509-10.
223. Nyaku AN, Williams LM, Galvin SR. Comparison of HIV Testing Uptake in an Urban Academic Emergency Department Using Different Testing Assays and Support Systems. AIDS patient care and STDs. 2016;30(4):166-9.
224. Obi A, Jones C, Pizzo E, Vera J, Hart C, Mackie N, et al. CD4 point-of-care testing improves patient satisfaction and reduces recalls to clinic. HIV Medicine. 2013;14:1-2.
225. O'Byrne P, MacPherson P, Ember A, Grayson M-O, Bourgault A. Overview of a gay men's STI/HIV testing clinic in Ottawa: clinical operations and outcomes. Canadian journal of public health = Revue canadienne de sante publique. 2014;105(5):e389-94.
226. O'Byrne P, MacPherson P, Roy M, Kitson C. Overviewing a Nurse-Led, Community-Based HIV PEP Program: Applying the Extant Literature in Frontline Practice. Public health nursing (Boston, Mass). 2015;32(3):256-65.
227. O'Byrne P, Phillips JC, Campbell B, Reynolds A, Metz G. Express testing in STI clinics: extant literature and preliminary implementation data. Applied nursing research : ANR. 2016;29:177-87.
228. O'Connor J, Vjecha MJ, Phillips AN, Angus B, Cooper D, Grinsztejn B, et al. Effect of immediate initiation of antiretroviral therapy on risk of severe bacterial infections in HIV-positive people with CD4 cell counts of more than 500 cells per µL: secondary outcome results from a randomised controlled trial. The lancet HIV. 2017;4(3):e105-e12.
229. Ojikutu B, Holman J, Kunches L, Landers S, Perlmutter D, Ward M, et al. Interdisciplinary HIV care in a changing healthcare environment in the USA. AIDS care. 2014;26(6):731-5.
230. Olding M, Enns B, Panagiotoglou D, Shoveller J, Harrigan PR, Barrios R, et al. A historical review of HIV prevention and care initiatives in British Columbia, Canada: 1996-2015. Journal of the International AIDS Society. 2017;20(1):21941.
231. Ong E, Brady M, Larbalestier N, Sonecha S, Bruton J, Weston R, et al. Ensuring implementation of BHIVA guidelines and pathways in HIV case management: An integrated care solution. HIV Medicine. 2013;14:20-1.
232. Pai NP, Sharma J, Shivkumar S, Pillay S, Vadnais C, Joseph L, et al. Supervised and unsupervised self-testing for HIV in high- and low-risk populations: a systematic review. PLoS Medicine. 2013;10(4):NA.
233. Pai NP, Wilkinson S, Deli-Houssein R, Vijh R, Vadnais C, Behlim T, et al. Barriers to Implementation of Rapid and Point-of-Care Tests for Human Immunodeficiency Virus Infection: Findings From a Systematic Review (1996-2014). Point of care. 2015;14(3):81-7.
234. Palar K, Napoles T, Hufstedler LL, Seligman H, Hecht FM, Madsen K, et al. Comprehensive and Medically Appropriate Food Support Is Associated with Improved HIV and Diabetes Health. Journal of urban health : bulletin of the New York Academy of Medicine. 2017;94(1):87-99.
235. Pandor A, Kaltenthaler E, Higgins A, Lorimer K, Smith S, Wylie K, et al. Sexual health risk reduction interventions for people with severe mental illness: a systematic review. BMC public health. 2015;15:138.
236. Paquette D, Schanzer D, Guo H, Gale-Rowe M, Wong T. The impact of HIV treatment as prevention in the presence of other prevention strategies: Lessons learned from a review of mathematical models set in developed countries. Canadian Journal of Infectious Diseases and Medical Microbiology. 2013;24:87A.
237. Parchure R, Kulkarni V, Kulkarni S, Gangakhedkar R. Pattern of linkage and retention in HIV care continuum among patients attending referral HIV care clinic in private sector in India. AIDS care. 2015;27(6):716-22.
238. Parsons J. Alcohol and HIV medication adherence: Takingan intervention from efficacy to effectiveness. Alcoholism: Clinical and Experimental Research. 2014;38:358A.
239. Parsons JT, Rendina HJ, Moody RL, Gurung S, Starks TJ, Pachankis JE. Feasibility of an Emotion Regulation Intervention to Improve Mental Health and Reduce HIV Transmission Risk Behaviors for HIV-Positive Gay and Bisexual Men with Sexual Compulsivity. AIDS and behavior. 2017;21(6):1540-9.
240. Peter T, Ellenberger D, Kim AA, Boeras D, Messele T, Roberts T, et al. Early antiretroviral therapy initiation: access and equity of viral load testing for HIV treatment monitoring. The Lancet Infectious Diseases. 2017;17(1):e26-e9.
241. Peter T, Zeh C, Katz Z, Elbireer A, Alemayehu B, Vojnov L, et al. Scaling up HIV viral load - lessons from the large-scale implementation of HIV early infant diagnosis and CD4 testing. Journal of the International AIDS Society. 2017;20 Suppl 7.
242. Philbin MM, Tanner AE, DuVal A, Ellen JM, Kapogiannis B, Fortenberry JD. Understanding Care Linkage and Engagement Across 15 Adolescent Clinics: Provider Perspectives and Implications for Newly HIV-Infected Youth. AIDS education and prevention : official publication of the International Society for AIDS Education. 2017;29(2):93-104.
243. Philbin MM, Tanner AE, DuVal A, Ellen JM, Xu J, Kapogiannis B, et al. HIV Testing, Care Referral, and Linkage to Care Intervals Affect Time to Engagement in Care for Newly Diagnosed HIV-Infected Adolescents in 15 Adolescent Medicine Clinics in the United States. Journal of acquired immune deficiency syndromes (1999). 2016;72(2):222-9.
244. Phillips T, McNairy ML, Zerbe A, Myer L, Abrams EJ. Implementation and Operational Research: Postpartum Transfer of Care Among HIV-Infected Women Initiating Antiretroviral Therapy During Pregnancy. Journal of acquired immune deficiency syndromes (1999). 2015;70(3):e102-9.
245. Pinto RM, Witte SS, Filippone PL, Choi CJ, Wall M. Policy Interventions Shaping HIV Prevention: Providers' Active Role in the HIV Continuum of Care. Health education & behavior : the official publication of the Society for Public Health Education. 2018:1090198118760681.
246. Pizzo E, Jones C, Obi A, Hart C, Vera J, Mackie N, et al. Can we justify use of a CD4 point-of-care test in a time of austerity? HIV Medicine. 2013;14:13.
247. PlusNews.Mental health blind spot limits MSM HIV interventions. PlusNews. 2013:NA.
248. Prendergast AJ, Essajee S, Penazzato M. HIV and the Millennium Development Goals. Archives of disease in childhood. 2015;100 Suppl 1:S48-52.
249. Purnomo J, Coote K, Mao L, Fan L, Gold J, Ahmad R, et al. Using eHealth to engage and retain priority populations in the HIV treatment and care cascade in the Asia-Pacific region: A systematic review of literature. BMC Infectious Diseases. 2018;18(1).
250. Quinlivan EB, Messer LC, Adimora AA, Roytburd K, Bowditch N, Parnell H, et al. Experiences with HIV testing, entry, and engagement in care by HIV-infected women of color, and the need for autonomy, competency, and relatedness. AIDS patient care and STDs. 2013;27(7):408-15.
251. Qvist T, Cowan SA, Graugaard C, Helleberg M. High linkage to care in a community-based rapid HIV testing and counseling project among men who have sex with men in Copenhagen. Sexually transmitted diseases. 2014;41(3):209-14.
252. Rabkin M, Fouad FM, El-Sadr WM. Addressing chronic diseases in protracted emergencies: Lessons from HIV for a new health imperative. Global Public Health. 2018;13(2):227-33.
253. Read TR HJ, Bradshaw CS, Morrow A, Grulich AE, Fairley CK, Chen MY. Provision of rapid HIV tests within a health service and frequency of HIV testing among men who have sex with men: randomised controlled trial. BMJ (clinical research ed). 2013;347:f5086.
254. Rebchook G, Keatley J, Contreras R, Perloff J, Molano LF, Reback CJ, et al. The Transgender Women of Color Initiative: Implementing and Evaluating Innovative Interventions to Enhance Engagement and Retention in HIV Care. American journal of public health. 2017;107(2):224-9.
255. 256. Reisner SL, Hughto JMW, Pardee DJ, Kuhns L, Garofalo R, Mimiaga MJ. LifeSkills for Men (LS4M): Pilot Evaluation of a Gender-Affirmative HIV and STI Prevention Intervention for Young Adult Transgender Men Who Have Sex with Men. Journal of urban health : bulletin of the New York Academy of Medicine. 2016;93(1):189-205.
256. Restall G, Sullivan T, Carnochan T, Etcheverry E, Roger K, Roddy P. A service delivery model for addressing activity and social participation needs of people living with HIV. Open Journal of Occupational Therapy. 2017;5(2):NA.
257. Reveles KR, Juday TR, Labreche MJ, Mortensen EM, Koeller JM, Seekins D, et al. Comparative value of four measures of retention in expert care in predicting clinical outcomes and health care utilization in HIV patients. PloS one. 2015;10(3):e0120953.
258. Reyes-Urueña J, Breveglieri M, Furegato M, Fernàndez-López L, Agusti C, Casabona J. Heterogeneity of community-based voluntary, counselling and testing services for HIV in Europe: the HIV-COBATEST survey. International journal of STD & AIDS. 2017;28(1):28-38.
259. Reyes-Urueña J, Fernàndez-López L, Force L, Daza M, Agustí C, Casabona J. Level of impact on the public health of universal human immunodeficiency virus screening in an Emergency Department. Enfermedades Infecciosas y Microbiologia Clinica. 2017;35(7):434-7.
260. Righetti A, Prinapori R, Nulvesu L, Fornoni L, Viscoli C, Di BA. Transitioning HIV-infected children and adolescents into adult care: An Italian real-life experience. J Assoc Nurses AIDS Care. 2015;26(5):652-9.
261. Rios-Ellis B, Bird M, Garcia-Vega M, Angel M, Valenzuela O, Ortega D, et al. Salud a la Vida: Developing HIV and hepatitis C prevention through community collaboration between a Hispanic-serving institution and its surrounding community. Journal of the International AIDS Society. 2016;19:22-3.
262. Risher K, Mayer KH, Beyrer C. HIV treatment cascade in MSM, people who inject drugs, and sex workers. Current opinion in HIV and AIDS. 2015;10(6):420-9.
263. Risher KA, Kapoor S, Daramola AM, Paz-Bailey G, Skarbinski J, Doyle K, et al. Challenges in the Evaluation of Interventions to Improve Engagement Along the HIV Care Continuum in the United States: A Systematic Review. AIDS and behavior. 2017;21(7):2101-23.
264. Rodkjaer LO, Laursen T, Seeberg K, Drouin M, Johansen H, Dyrehave C, et al. The Effect of a Mind-Body Intervention on Mental Health and Coping Self-Efficacy in HIV-Infected Individuals: A Feasibility Study. Journal of alternative and complementary medicine (New York, NY). 2017;23(5):326-30.
265. Rodkjaer LO LT, Seeberg K, Drouin M, Johansen H, Dyrehave C, Honge BL, Ostergaard L. The Effect of a Mind-Body Intervention on Mental Health and Coping Self-Efficacy in HIV-Infected Individuals: a Feasibility Study. Journal of alternative and complementary medicine (new york, NY). 2017;23(5):326-30.
266. Rodriguez HR, Dobalian A. Provider and Administrator Experiences With Providing HIV Treatment and Prevention Services in Rural Areas. AIDS education and prevention : official publication of the International Society for AIDS Education. 2017;29(1):77-91.
267. Romero LM, Olaiya O, Hallum-Montes R, Varanasi B, Mueller T, House LD, et al. Efforts to Increase Implementation of Evidence-Based Clinical Practices to Improve Adolescent-Friendly Reproductive Health Services. Journal of Adolescent Health. 2017;60(3):S30-S7.
268. Rosales-Statkus ME, de la Fuente L, Fernández-Balbuena S, Figueroa C, Fernàndez-López L, Hoyos J, et al. Approval and potential use of over-the-counter HIV self-tests: the opinion of participants in a street based HIV rapid testing program in Spain. AIDS and behavior. 2015;19(3):472-84.
269. Rukh S, Khurana R, Mickey T, Anderson L, Velasquez C, Taylor M. Chlamydia and gonorrhea diagnosis, treatment, personnel cost savings, and service delivery improvements after the implementation of express sexually transmitted disease testing in Maricopa County, Arizona. Sexually transmitted diseases. 2014;41(1):74-8.
270. Saberi P, Johnson MO. Moving toward a novel and comprehensive behavioral composite of engagement in HIV care. AIDS care. 2015;27(5):660-4.
271. Sangaramoorthy T, Jamison AM, Dyer TV. HIV Stigma, Retention in Care, and Adherence Among Older Black Women Living With HIV. The Journal of the Association of Nurses in AIDS Care : JANAC. 2017;28(4):518-31.
272. Santella AJ, Krishnamachari B, Davide SH, Cortell M, Furnari W, Watts B, et al. Dental hygienists' knowledge of HIV, attitudes towards people with HIV and willingness to conduct rapid HIV testing. International journal of dental hygiene. 2013;11(4):287-92.
273. Saritha K, Himabindu P, Shobitha GL, Vasudha M. A retrospective 5 year study of PPTCT programme at tertiary care centre. Journal of Evolution of Medical and Dental Sciences. 2015;4(46):8000(9).
274. Schuettfort G, De Leuw P, Haberl A, Stephan C, Nguyen M. Implementation of pharmacists to an interdisciplinary care provider team for people living with HIV-6 month interims analysis. Clinical Therapeutics. 2017;39(8):e46-e7.
275. Schwartz CE, Finkelstein JA, Rapkin BD. Appraisal assessment in patient-reported outcome research: methods for uncovering the personal context and meaning of quality of life. Quality of Life Research. 2017;26(3):545(10).
276. Schwartz RP, Stitzer ML, Feaster DJ, Korthuis PT, Alvanzo AA, Winhusen TM, Donnard L, Snead N, Metsch LR. HIV rapid testing in drug treatment: comparison across treatment modalities. Journal of Substance Abuse Treatment. 2013;44(4):369(6).
277. Scott L, Da Silva P, Boehme CC, Stevens W, Gilpin CM. Diagnosis of opportunistic infections: HIV co-infections-tuberculosis. Current Opinion in HIV and AIDS. 2017;12(2):129-38.
278. Seeman MV. The role of mental health services in addressing HIV infection among women with serious mental illness. Psychiatric Services. 2015;66(9):966-74.
279. Seery P, Freeman A, Foster C. Referral to Community Adolescent Mental Health Services (CAMHS) in a perinatally infected adolescent cohort. HIV Medicine. 2017;18:29.
280. Sekhon P, Corredor C, Resinenete J, Quraishi A, Dhairyawan R, Soni S. Outreach initiatives encourage HIV testing in hard-to-reach communities. HIV Medicine. 2014;15:106-7.
281. Selway D, Liu K, Sonecha S, Sullivan A, Asboe D. A review of a quality improvement intervention to increase documentation of flu vaccination status in a large HIV unit. HIV Medicine. 2016;17:70.
282. Selway D LK, Sonecha S, Sullivan A, Asboe D. A review of a quality improvement intervention to increase documentation of flu vaccination status in a large HIV unit. HIV medicine Conference: 22nd annual conference of the british HIV association, BHIVA 2016 United kingdom Conference start: 20160419 Conference end: 20160422. 2016;17:70.
283. Seng EK, Lovejoy TI. Reliability and Validity of a Treatment Fidelity Assessment for Motivational Interviewing Targeting Sexual Risk Behaviors in People Living with HIV/AIDS. Journal of Clinical Psychology in Medical Settings. 2013;20(4):440(9).
284. Senn TE, Braksmajer A, Coury-Doniger P, Urban MA, Carey MP. Mobile technology use and desired technology-based intervention characteristics among HIV+ Black men who have sex with men. AIDS Care - Psychological and Socio-Medical Aspects of AIDS/HIV. 2017;29(4):423-7.
285. Shahi R, Kiani G, Alimohammadi A, Raycraft T, Singh A, Hakobyan S, et al. HIV support group within a multidisciplinary healthcare delivery model as a treatment strategy for people who inject drugs. Journal of the International AIDS Society. 2016;19:62.
286. Shindler S, Ringlein M. Oh baby! evaluation of a prenatal group for women living with HIV. Canadian Journal of Infectious Diseases and Medical Microbiology. 2013;24:34A.
287. Shoemaker SJ, Curran GM, Swan H, Teeter BS, Thomas J. Application of the Consolidated Framework for Implementation Research to community pharmacy: A framework for implementation research on pharmacy services. Research in social & administrative pharmacy : RSAP. 2017;13(5):905-13.
288. Silverberg MJ, Leyden W, Lin H, Qin L, Achenbach CJ, D'Souza G, et al. Earlier versus delayed antiretroviral therapy initiation and risk of cancer. Topics in Antiviral Medicine. 2017;25(1):248s.
289. Simeone CA, Seal SM, Savage C. Implementing HIV Testing in Substance Use Treatment Programs: A Systematic Review. The Journal of the Association of Nurses in AIDS Care : JANAC. 2017;28(2):199-215.
290. Simonsen KA, Shaikh RA, Earley M, Foxall M, Boyle C, Islam KM, et al. Rapid HIV Screening in an Urban Jail: How Testing at Exit With Linkage to Community Care Can Address Perceived Barriers. The journal of primary prevention. 2015;36(6):427-32.
291. Simonsen SE, Kepka D, Thompson J, Warner EL, Snyder M, Ries KM. Preventive health care among HIV positive women in a Utah HIV/AIDS clinic: A retrospective cohort study. BMC Women's Health. 2014;14(1).
292. Singh A, Alimohammadi A, Raycraft T, Kiani G, Shahi R, Conway B. Utilizing the Ottawa charter in a multidisciplinary setting as a harm reduction strategy in people who inject drugs. Hepatology International. 2017;11(1):S559.
293. Skinta MD, Lezama M, Wells G, Dilley JW. Acceptance and Compassion-Based Group Therapy to Reduce HIV Stigma. Cognitive and Behavioral Practice. 2015;22(4):481-90.
294. Smith S. Spread the word on mental health aid. Edmonton Journal. 2017.
295. Sprague C, Simon SE. Understanding HIV care delays in the US South and the role of the social-level in HIV care engagement/retention: a qualitative study. International journal for equity in health. 2014;13:28.
296. Srivastava M, Sullivan D, Phelps BR, Modi S, Broyles LN. Boosting ART uptake and retention among HIV-infected pregnant and breastfeeding women and their infants: The promise of innovative service delivery models. Journal of the International AIDS Society. 2018;21(1).
297. St Lawrence JS, Kelly JA, Dickson-Gomez J, Owczarzak J, Amirkhanian YA, Sitzler C. Attitudes Toward HIV Voluntary Counseling and Testing (VCT) Among African American Men Who Have Sex With Men: Concerns Underlying Reluctance to Test. AIDS education and prevention : official publication of the International Society for AIDS Education. 2015;27(3):195-211.
298. Steen R, Wheeler T, Gorgens M, Mziray E, Dallabetta G. Feasible, Efficient and Necessary, without Exception - Working with Sex Workers Interrupts HIV/STI Transmission and Brings Treatment to Many in Need. PLoS ONE. 2015;10(10):NA.
299. Stockwell S, Dean G, Tweed M, Boyt T. Pro(TECT) service-engaging with male sex workers. Sexually Transmitted Infections. 2015;91:A86-A7.
300. Stricker SM, Fox KA, Baggaley R, Negussie E, de Pee S, Grede N, et al. Retention in care and adherence to ART are critical elements of HIV care interventions. AIDS and behavior. 2014;18:S465-S75.
301. Strömdahl S, Liljeros F, Thorson AE, Persson KI, Forsberg BC. HIV testing and prevention among foreign-born Men Who have Sex with Men: an online survey from Sweden. BMC public health. 2017;17(1):139.
302. Stuber M, Spence Gress C, Rodger D, Wong A. Clinic to community: Inter-professional collaboration in enhanced adherence to antiretroviral therapy. Canadian Journal of Infectious Diseases and Medical Microbiology. 2014;25:54A-5A.
303. Sullivan KA, Schultz K, Ramaiya M, Berger M, Parnell H, Quinlivan EB. Experiences of women of color with a nurse patient navigation program for linkage and engagement in HIV care. AIDS patient care and STDs. 2015;29 Suppl 1:S49-54.
304. Suthar AB, Ford N, Bachanas PJ, Wong VJ, Rajan JS, Saltzman AK, et al. Towards universal voluntary HIV Testing and counselling: a systematic review and meta-analysis of community-based approaches. PLoS Medicine. 2013;10(8):NA.
305. Suthar AB, Rutherford GW, Horvath T, Doherty MC, Negussie EK. Improving antiretroviral therapy scale-up and effectiveness through service integration and decentralization. AIDS. 2014;28(SUPPL. 2):S175-S85.
306. Swan H, O'Connell DJ, Visher CA, Martin SS, Swanson KR, Hernandez K. Improvements in Correctional HIV Services: A Case Study in Delaware. Journal of correctional health care : the official journal of the National Commission on Correctional Health Care. 2015;21(2):164-76.
307. Swendeman D, Ramanathan N, Baetscher L, Medich M, Scheffler A, Comulada WS, et al. Smartphone Self-Monitoring to support self-management among people living with HIV: Perceived benefits and theory of change from a mixed-methods randomized pilot study. Journal of Acquired Immune Deficiency Syndromes. 2015;69:S80-S91.
308. Tanner AE, Mann L, Song E, Alonzo J, Schafer K, Arellano E, et al. weCARE: A Social Media-Based Intervention Designed to Increase HIV Care Linkage, Retention, and Health Outcomes for Racially and Ethnically Diverse Young MSM. AIDS education and prevention : official publication of the International Society for AIDS Education. 2016;28(3):216-30.
309. Tanner AE, Philbin MM, Duval A, Ellen J, Kapogiannis B, Fortenberry JD. Youth friendly clinics: considerations for linking and engaging HIV-infected adolescents into care. AIDS care. 2014;26(2):199-205.
310. Tanser F, Bärnighausen T, Vandormael A, Dobra A. HIV treatment cascade in migrants and mobile populations. Current opinion in HIV and AIDS. 2015;10(6):430-8.
311. Taylor BS, Olender SA, Tieu H-V, Wilkin TJ. CROI 2016: Advances in Antiretroviral Therapy. Topics in antiviral medicine. 2016;24(1):59-81.
312. Tedaldi EM, Richardson JT, Debes R, Young B, Chmiel JS, Durham MD, et al. Retention in care within 1 year of initial HIV care visit in a multisite US cohort: who's in and who's out? Journal of the International Association of Providers of AIDS Care. 2014;13(3):232-41.
313. Tobin K, Davey-Rothwell MA, Nonyane BAS, Knowlton A, Wissow L, Latkin CA. RCT of an integrated CBT-HIV intervention on depressive symptoms and HIV risk. PLoS ONE. 2017;12(12):e0187180.
314. Tolou-Shams M, Dauria E, Conrad SM, Kemp K, Johnson S, Brown LK. Outcomes of a family-based HIV prevention intervention for substance using juvenile offenders. Journal of Substance Abuse Treatment. 2017;77:115-25.
315. Tran BX, Nguyen LH, Nguyen LP, Nguyen CT, Latkin CA. Methadone Maintenance Treatment Promotes Referral and Uptake of HIV Testing and Counselling Services amongst Drug Users and Their Partners. PLoS ONE. 2016;11(4):NA.
316. Tsai AC, Mimiaga MJ, Dilley JW, Hammer GP, Karasic DH, Charlebois ED, et al. Does effective depression treatment alone reduce secondary HIV transmission risk? Equivocal findings from a randomized controlled trial. AIDS and behavior. 2013;17(8):2765-72.
317. Tucker JD, Tso LS, Hall B, Ma Q, Beanland R, Best J, et al. Enhancing Public Health HIV Interventions: A Qualitative Meta-Synthesis and Systematic Review of Studies to Improve Linkage to Care, Adherence, and Retention. EBioMedicine. 2017;17:163-71.
318. Turner SD, Anderson K, Slater M, Quigley L, Dyck M, Guiang CB. Rapid point-of-care HIV testing in youth: a systematic review. The Journal of adolescent health : official publication of the Society for Adolescent Medicine. 2013;53(6):683-91.
319. Uebelacker LA, Weisberg RB, Herman DS, Bailey GL, Pinkston-Camp MM, Stein MD. Chronic Pain in HIV-Infected Patients: Relationship to Depression, Substance Use, and Mental Health and Pain Treatment. Pain Medicine. 2015;16(10):1870(12).
320. Underhill K, Morrow K, Holcomb R, Operario D, Mayer K. Behavior disclosure, access to healthcare, and HIV/ STI testing among male sex workers and other MSM in the us: Findings from a qualitative study on prep acceptability. Sexually Transmitted Diseases. 2014;41:S103.
321. Underhill K, Morrow KM, Colleran CM, Holcomb R, Operario D, Calabrese SK, et al. Access to healthcare, HIV/STI testing, and preferred pre-exposure prophylaxis providers among men who have sex with men and men who engage in street-based sex work in the US. PLoS ONE. 2014;9(11).
322. Vagenas P, Zelenev A, Altice FL, Di Paola A, Jordan AO, Teixeira PA, et al. HIV-infected men who have sex with men, before and after release from jail: the impact of age and race, results from a multi-site study. AIDS care. 2016;28(1):22-31.
323. Valle S, Pezzotti P, Floridia M, Pellegrini MG, Bernardi S, Puro V, et al. Percentage and determinants of missed HIV testing in pregnancy: a survey of women delivering in the Lazio region, Italy. AIDS care. 2014;26(7):899-906.
324. Venturelli S, McKenna W, Carder M, Ramzan F, Negedu O, Bailey AC, et al. START: How long does it take to start antiretroviral therapy (ART)? HIV Medicine. 2017;18:20.
325. Vermund SH, Mallalieu EC, Van Lith LM, Struthers HE. Health Communication and the HIV Continuum of Care. Journal of acquired immune deficiency syndromes (1999). 2017;74 Suppl 1:S1-S4.
326. Villar-Loubet OM, Illa L, Echenique M, Cook R, Messick B. Prenatal and Mental Health Care Among Trauma-Exposed HIV-Infected, Pregnant Women in the United States. Journal of the Association of Nurses in AIDS Care. 2014;25(1):S50-S61.
327. Vreeman RC, McCoy BM, Lee S. Mental health challenges among adolescents living with HIV. Journal of the International AIDS Society. 2017;20.
328. Wainberg MA, Hull MW, Girard P-M, Montaner JSG. Achieving the 90-90-90 target: incentives for HIV testing. The Lancet Infectious diseases. 2016;16(11):1215-6.
329. Wall KM, Canary L, Workowski K, Lockard A, Jones J, Sullivan P, et al. Acceptability of Couples' Voluntary HIV Testing Among HIV-infected Patients in Care and Their HIV-negative Partners in the United States. The open AIDS journal. 2016;10:1-13.
330. Weidle PJ, Lecher S, Botts LW, Jones L, Spach DH, Alvarez J, et al. HIV testing in community pharmacies and retail clinics: a model to expand access to screening for HIV infection. Journal of the American Pharmacists Association : JAPhA. 2014;54(5):486-92.
331. Wester C, Rebeiro PF, Shavor TJ, Shepherd BE, McGoy SL, Daley B, et al. The 2013 HIV Continuum of Care in Tennessee: Progress Made, but Disparities Persist. Public health reports (Washington, DC : 1974). 2016;131(5):695-703.
332. Whiteley LB, Brown LK, Swenson R, Kapogiannis BG, Harper GW. Disparities in mental health care among HIV-infected youth. Journal of the International Association of Physicians in AIDS Care. 2014;13(1):29-34.
333. Wilson PA, Valera P, Martos AJ, Wittlin NM, Muñoz-Laboy MA, Parker RG. Contributions of Qualitative Research in Informing HIV/AIDS Interventions Targeting Black MSM in the United States. Journal of sex research. 2016;53(6):642-54.
334. Wohl AR, Dierst-Davies R, Victoroff A, James S, Bendetson J, Bailey J, et al. Implementation and Operational Research: The Navigation Program: An Intervention to Reengage Lost Patients at 7 HIV Clinics in Los Angeles County, 2012-2014. Journal of Acquired Immune Deficiency Syndromes. 2016;71(2):e44-e50.
335. Wong VJ, Murray KR, Phelps BR, Vermund SH, McCarraher DR. Adolescents, young people, and the 90-90-90 goals: A call to improve HIV testing and linkage to treatment. AIDS. 2017;31:S191-S4.
336. Wouters K, Fransen K, Beelaert G, Kenyon C, Platteau T, Van Ghyseghem C, et al. Use of rapid HIV testing in a low threshold centre in Antwerp, Belgium, 2007–2012. International Journal of STD and AIDS. 2014;25(13):936-42.
337. Yu J, Appel P, Rogers M, Blank S, Davis C, Warren B, et al. Integrating intervention for substance use disorder in a healthcare setting: practice and outcomes in New York City STD clinics. The American journal of drug and alcohol abuse. 2016;42(1):32-8.
338. Zhou K, Walsh N, Fitzpatrick T, Kim JY, Lo Y-R, Tucker JD. Interventions to optimize retention in the chronic viral hepatitis screening, care and treatment cascade: A systematic review. Hepatology. 2015;62:496A.
339. Zimmermann R, Marcus U, Schäffer D, Leicht A, Wenz B, Nielsen S, et al. A multicentre sero-behavioural survey for hepatitis B and C, HIV and HTLV among people who inject drugs in Germany using respondent driven sampling. BMC public health. 2014;14:845.
