# Supplementary material for: A need for implementation science to optimise the use of evidence-based interventions in HIV care: A systematic literature review
Source: PLoS One. 2019 Aug 19;14(8):e0220060. doi: 10.1371/journal.pone.0220060 (PMC6699703; doi:10.1371/journal.pone.0220060)
Supplement: S1 Fig — Objective: The ViiV Healthcare Positive Pathways initiative was established to define best practice and interventions that can effectively close the gaps in the treatment continuum. Development methodology: The methodology used to develop the compendium was divided in the three phases. Phase 1 literature search: To develop a baseline of the scope and range of activities of current evidence-based practice, a literature search of key published evidence was undertaken from 2010 to 2016. This was a non-systematic literature review using PubMed and Google Scholar as research databases and focusing on geography in the scope of the project. We reviewed a range of articles and journals, from internationally approved guidelines (WHO, IAPAC, CDC, ECDC, NICE and DHHS) for evidence-based practice care and management as well as reviews and observational studies in single centres. An initial list of 66 EBIs was developed from this first phase of the work. Phase 2 site visits: In the next phase of the work, we visited eight established centres delivering HIV care to observe current practice and establish the extent and scope of EBIs in use. Centres were identified through the ViiV Healthcare network and selected based on their commitment to UNAIDS 90-90-90 targets and willingness to participate in the initiative including the dissemination of findings through data publication in a relevant peer-reviewed journal. Centres were in Western Europe and North America. Performance data relating to the 90-90-90 targets, were collected and we conducted over 100 interviews across a wide range of stakeholders. An interview guide was created to facilitate structured collection of quantitative and qualitative data on HIV care and management across the care continuum. This included centre and community involvement in HIV awareness and prevention through to disease diagnosis, linkage to and retention in care and clinical management and follow-up. Interview participants were selected by the lead e [file pone.0220060.s001.docx]

S1 Fig. Positive Pathways Initiative: Development of the Compendium of EBIs. Objective: The ViiV Healthcare Positive Pathways Initiative was established to define best practice and interventions that can effectively close the gaps in the treatment continuum. Development methodology: The methodology used to develop the compendium was divided in the three phases. *Phase 1 literature search*: To develop a baseline of the scope and range of activities of current evidence-based practice, a literature search of key published evidence was undertaken from 2010 to 2016. This was a non-systematic literature review using PubMed and Google Scholar as research databases and focusing on geography in the scope of the project. We reviewed a range of articles and journals, from internationally approved guidelines (WHO, IAPAC, CDC, ECDC, NICE and DHHS) for evidence-based practice care and management as well as reviews and observational studies in single centres. An initial list of 66 EBIs was developed from this first phase of the work. *Phase 2 site visits*: In the next phase of the work, we visited eight established centres delivering HIV care to observe current practice and establish the extent and scope of EBIs in use. Centres were identified through the ViiV Healthcare network and selected based on their commitment to UNAIDS 90-90-90 targets and willingness to participate in the initiative including the dissemination of findings through data publication in a relevant peer-reviewed journal. Centres were in Western Europe and North America. Performance data relating to the 90-90-90 targets, were collected and we conducted over 100 interviews across a wide range of stakeholders. An interview guide was created to facilitate structured collection of quantitative and qualitative data on HIV care and management across the care continuum. This included centre and community involvement in HIV awareness and prevention through to disease diagnosis, linkage to and retention in care and clinical management and follow-up. Interview participants were selected by the lead experts within each centre. Selection criteria were based on a participant’s input and level of experience in care and management of HIV patients. We observed HIV care being delivered in a wide range of care settings that included Specialist HIV Centres, Infectious Disease Departments, Sexual Health Clinics and Primary Care Centres, each with specific features. Observations at site visits were cross-referenced against the findings of our secondary literature review to help develop the compendium and HIV care and management assessment questionnaire. *Phase 3 development of compendium*: In the final phase to develop the compendium three advisory boards with 12 experts were held to test and prioritise the key intervention categories and interventions resulting from the first and second phase. Participants included experts from the participating centre, a health economist, patient advocacy group representative and a healthcare systems manager. Within this programme, EBIs were thematically grouped into six categories and prioritised in terms of impact and practicality for implementation leading to the establishment of a final compendium of 21 EBIs. From the compendium of 21 interventions, 12 were prioritized by an expert panel across six key themes of current HIV practice (interventions shaded under each of the six themes). Prioritization was based on a consideration of feasibility/perceived ease for care centres to trial the EBI. These EBIs are expected to be more widely used, investigated and reported. These 12 EBIs from the Positive Pathways initiative were included in the scope of the review.
